# A Hierarchical Semantic Knowledge-Distilled Belief Rule Base for Interpretable Fault Diagnosis

**DOI:** 10.3390/s26206374

**Published:** 2026-10-09

**Authors:** Zhiying Fan, Yuanyuan Qu, Wei He

**Affiliations:** 1 School of Computer Science and Information Engineering, Harbin Normal University, Harbin 150025, China; 2024300703@stu.hrbnu.edu.cn; 2 School of Information Engineering, Heilongjiang Agricultural Engineering Vocational and Technical University, Harbin 150080, China; candyuan@live.cn

**Keywords:** fault diagnosis, belief rule base, knowledge distillation, interpretable artificial intelligence, rotating machinery

## Abstract

Fault diagnosis is essential for the safe and reliable operation of industrial equipment. Deep learning models provide strong fault recognition capabilities, but offer limited transparency, while belief rule bases (BRBs) provide explicit reasoning, but face challenges in representing complex fault relationships and meaningful intermediate states. Existing knowledge distillation methods mainly focus on final outputs and do not explicitly align intermediate semantic states and diagnostic paths. Moreover, conventional classification metrics do not directly evaluate hierarchical reasoning consistency. To address these issues, this paper proposes a hierarchical semantic knowledge-distilled belief rule base (HSD-BRB). Expert-guided semantic modeling assigns explicit meanings to intermediate states, while output-, state-, and path-level knowledge distillation transfers teacher knowledge to the corresponding levels of the BRB. An expert-deviation constraint further preserves the initialized rule knowledge, and a consistency evaluation mechanism quantifies state- and path-level alignment. Experiments on the Southeast University bearing and gearbox datasets achieve mean Accuracies of 95.76% and 96.81% and Macro-F1 scores of 0.9575 and 0.9680, respectively. In the gearbox small-sample setting, the BRB is optimized using 90 labelled samples, while the teacher uses the full 890-sample training partition. The results show improved state- and path-level consistency together with traceable semantic states, rule activations, and final fault beliefs. Overall, HSD-BRB provides an interpretable framework for transferring neural knowledge into hierarchical rule-based fault diagnosis.

## 1. Introduction

Fault diagnosis plays an important role in maintaining the safe operation of industrial equipment, improving maintenance efficiency, and reducing maintenance costs. Bearings, gearboxes, and other rotating components often operate under varying loads and speeds. Their vibration signals are nonlinear, nonstationary, and subject to substantial uncertainty, and different fault conditions may produce similar responses [1,2,3,4]. A useful diagnostic model should therefore provide not only accurate predictions, but also a clear account of how those predictions are obtained. Recent reviews have emphasized that interpretability is becoming an important requirement for trustworthy fault diagnosis, with ante-hoc approaches offering a more direct connection between model reasoning and domain knowledge [5].

For the present discussion, related diagnostic methods are organized by their main source of information: data-driven representation learning, explicit rule-based reasoning, and data–knowledge fusion. These are overlapping research strands rather than mutually exclusive categories. Representative original studies include deep transfer learning for machinery diagnosis [6], rule induction for rotating machinery [7], and interpretable hierarchical BRB modeling [8].

Data-driven methods learn fault-related representations from measured signals or extracted features. Shao et al. combine feature learning with transfer learning to support machinery diagnosis. Such models can capture nonlinear class boundaries, but their predictions do not directly expose a sequence of named semantic states and belief-rule activations. Rule-based approaches instead express decisions through explicit antecedent–consequent relationships. For example, Dou et al. combine empirical mode decomposition, dimensionless features, decision-table rule induction, and a rule-matching strategy. This provides an inspectable diagnostic procedure, although its rule construction differs from teacher-guided learning of a hierarchical belief distribution.

Data–knowledge fusion combines structured prior knowledge with parameters learned from observations. BRB inference is a relevant example: the RIMER framework represents uncertain rule consequents through belief degrees and combines activated rules using evidential reasoning [9]. The research strand most relevant to HSD-BRB is therefore interpretable BRB learning, particularly hierarchical structures and teacher-guided parameter optimization, rather than neural classification alone.

Several studies have addressed the construction and interpretability of BRB models. Cao et al. introduce an approximate BRB to support interpretable large-scale expert-system modeling [10]. Hu et al. combine hierarchical BRBs with feature selection for imbalanced multiclass problems [11]. Yang et al. develop a multilayer tree-structured BRB for complex-problem modeling [12]. Cao et al.’s interpretable hierarchical model explicitly addresses readability and rule consistency through construction and optimization strategies. These studies establish that hierarchy alone is not a new contribution; the question considered here is how supervision from a neural teacher can be associated with the intermediate states and rule activations of that hierarchy.

Preserving expert meaning during optimization is also an established concern. Ming et al. construct a BRB using a probability table and incorporate interpretability constraints to limit deviation from expert judgment and over-optimization [13]. This motivates treating expert preservation separately from predictive accuracy. HSD-BRB follows this general concern but evaluates it together with explicit state- and path-level teacher–student alignment, rather than treating a low classification error as sufficient evidence of consistent reasoning.

Knowledge distillation supplies a complementary mechanism for learning from another model. Hinton et al. transfer softened class-output distributions [14]; FitNets uses intermediate teacher representations as learning hints [15]; and relational knowledge distillation matches relationships among examples [16]. Thus, distillation is not restricted to final outputs. However, feature or sample-relation alignment does not by itself specify how supervision should correspond to named BRB states or combinations of activated rules. Direct distillation into a BRB is also not new: Zeng et al. identify discrepant samples and rectify BRB predictions by weighted alignment with machine-learning outputs, with interpretable contribution analysis [17]. The present work instead focuses on explicit alignment at multiple levels of a semantic BRB hierarchy.

Table 1 motivates three linked research questions: how to define a correspondence between teacher supervision and hierarchical BRB states; how to align both those states and the top-level rule-activation distribution; and how to assess this alignment separately from classification accuracy and preservation of expert rules. The semantic targets used here are operational definitions based on feature groups and training-set reference values, not independently verified physical states. Accordingly, agreement with these targets is distinguished from semantic correctness.

To address these questions, HSD-BRB makes the following contributions:

1. An expert-guided hierarchical semantic modeling method is developed to organize multiple attributes into meaningful semantic subspaces and construct the associated hierarchical BRB.
2. A hierarchical distillation mechanism is introduced to transfer fault-output, semantic-state, and rule-related knowledge to the corresponding levels of the BRB.
3. A diagnosis-chain explanation and consistency evaluation method is established to analyze the state distributions, rule activations, and final fault beliefs, and to quantify how well the BRB preserves the teacher’s diagnostic knowledge.

Experiments on measured bearing and gearbox vibration data evaluate these contributions through internal ablations, external baselines, sensitivity and significance analyses, and robustness tests. The contribution of HSD-BRB is an inspectable multilevel transfer mechanism that aligns teacher knowledge with the semantic states and rule paths of a hierarchical BRB.

The remainder of this paper is organized as follows. Section 2 introduces the hierarchical belief rule base and formulates the fault diagnosis problem. Section 3 presents HSD-BRB, including hierarchical semantic modeling, knowledge distillation, and traceable diagnosis. Section 4 reports the case studies and experimental results. Section 5 concludes the paper.

## 2. HBRB and Problem Description

Section 2.1 introduces the basic principles and inference procedure of the hierarchical belief rule base (HBRB). Section 2.2 formulates the rotating machinery fault diagnosis problem and gives its mathematical description.

### 2.1. Hierarchical Belief Rule Base

A belief rule base (BRB) uses IF–THEN rules with belief distributions to describe uncertain relationships between input attributes and output conclusions [9,18]. The k-th belief rule consists of the rule antecedents, the consequent belief distribution, and the rule weight, and can be expressed as

(1) $$\begin{array}{c} R_{k}:\;\mathrm{If}\;x_{1}\;\mathrm{is}\;A_{1}^{k}\wedge \ldots \wedge x_{M}\;\mathrm{is}\;A_{M}^{k},\;\mathrm{Then}\;{\left\{\left(D_{n},\beta_{n,k}\right)\right\}}_{n=1}^{N},\;\mathrm{with}\;\mathrm{rule}\;\mathrm{weight}\;\theta_{k}. \end{array}$$

Here, $x_{m}$ denotes the *m*-th input attribute and $A_{m}^{k}$ denotes the corresponding reference state of $x_{m}$ in rule $R_{k}$. $D_{n}$ represents the *n*-th output state, $\beta_{n,k}$ is the belief degree assigned to $D_{n}$ by rule $R_{k}$, and $\theta_{k}$ is the rule weight. The relative importance of the input attributes is described by the attribute-weight vector $\delta =[\delta_{1},\ldots ,\delta_{M}]$.

When the sum of the consequent belief degrees is equal to one, the consequent information is complete. If this sum is less than one, the unassigned belief represents the incompleteness of the rule.

Each rule also has a rule weight, which describes its relative importance during inference. Similarly, each input attribute has an attribute weight that describes its relative influence on rule activation.

For a flat BRB with multiple input attributes, the combinations of the reference states of all attributes jointly form the rule antecedents. If the *m*-th attribute contains $J_{m}$ reference states, the total number of rules in the complete rule base is given by

(2) $$\begin{array}{c} L_{\mathrm{flat}}=\prod_{m=1}^{M}J_{m}. \end{array}$$

**Figure 1:**
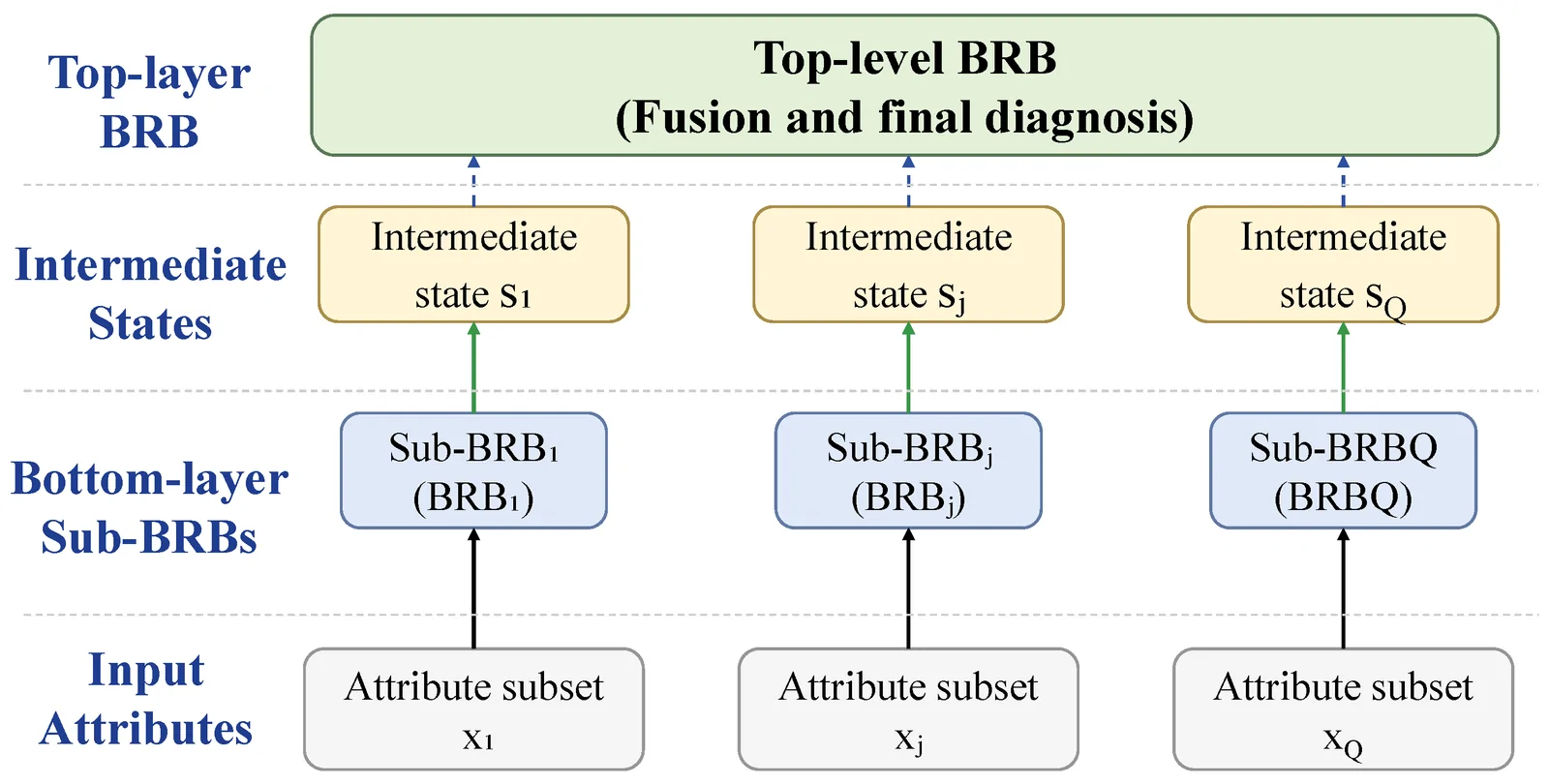
The hierarchical structure of HBRB.

As the number of input attributes and their reference states increases, the size of a complete rule base grows rapidly. A hierarchical belief rule base (HBRB) decomposes the overall reasoning task into several interrelated low-dimensional BRBs and connects different reasoning levels through intermediate states. This allows multi-attribute information to be represented and fused in a hierarchical manner. The hierarchical structure of HBRB is illustrated in Figure 1.

Let $x=[x_{1},\ldots ,x_{Q}]$ denote the input partitioned into *Q* attribute groups. Sub-BRB *q* produces $s_{q}=\left[\beta_{q,1}^{s},\ldots ,\beta_{q,H_{q}}^{s}\right]$, where $H_{q}$ is its number of intermediate states. The hierarchical mapping is

(3) $$s_{q}={\mathrm{BRB}}_{q}\left(x_{q}\right),\;\beta^{F}={\mathrm{BRB}}_{T}\left(s_{1},\ldots ,s_{Q}\right).$$

The final belief vector $\beta^{F}=\left[\beta_{1}^{F},\ldots ,\beta_{C}^{F}\right]$ assigns beliefs to the fault states $F=\{F_{1},\ldots ,F_{C}\}$, and the predicted class is $\hat{y}=\arg\max_{c}\beta_{c}^{F}$. Passing complete intermediate distributions preserves uncertainty for upper-level aggregation. For example, $s_{q}=\left[0.10,0.25,0.65\right]$ retains the beliefs in all three states instead of passing only the most likely state.

If sub-BRB *q* contains $M_{q}$ attributes with $J_{q,m}$ reference states, its rule count is $L_{q}=\prod_{m=1}^{M_{q}}J_{q,m}$. The top-level rule count is $L_{T}=\prod_{q=1}^{Q}H_{q}$, giving

(4) $$L_{\mathrm{HBRB}}=\sum_{q=1}^{Q}L_{q}+L_{T}.$$

This decomposition represents local attribute relationships through low-dimensional sub-BRBs and combines their intermediate beliefs at the top level, providing a traceable connection between the inputs and the diagnosis.

### 2.2. Problem Description

Let the fault diagnosis dataset be defined as $D={\left\{\left(x_{i},y_{i}\right)\right\}}_{i=1}^{N}$, $x_{i}\in R^{M}$, $y_{i}\in \left\{1,2,\ldots ,C\right\}$, where *N*, *M*, and *C* denote the numbers of samples, input attributes, and fault classes, respectively. The HBRB distributes the input attributes among several sub-BRBs and uses the output of each sub-BRB as an input to the top-level BRB. Based on this hierarchical structure, the following three issues are considered in this study.

**Problem** **1.** *Semantic ambiguity in the hierarchical organization. Suppose that the i-th sample is divided into Q attribute subsets: $x_{i}=\left[x_{i,1},x_{i,2},\ldots ,x_{i,Q}\right],s_{i,q}={\mathrm{BRB}}_{q}\left(x_{i,q}\right).$*

The HBRB provides the connection between the sub-BRBs and the top-level BRB. However, the meaning of the intermediate state $s_{i,q}$ is not determined by the hierarchical structure itself. If related attributes are assigned to different submodules, or if the intermediate states are not associated with the corresponding fault mechanisms, the sub-BRB outputs may be treated merely as numerical variables during top-level fusion. In this case, although the HBRB has a hierarchical form, its intermediate reasoning process is difficult to interpret. The first problem is therefore how to establish a clear semantic relationship between attribute organization and intermediate states.

**Problem** **2.** *Inconsistency between output imitation and internal inference. Let the fault probability distribution produced by the teacher model be $p_{i}^{T}=\left[p_{i,1}^{T},p_{i,2}^{T},\ldots ,p_{i,C}^{T}\right],$ and let the final fault belief distribution produced by the HBRB be $\beta_{i}^{S}=\left[\beta_{i,1}^{S},\beta_{i,2}^{S},\ldots ,\beta_{i,C}^{S}\right].$*

Output-level knowledge distillation constrains the difference between $p_{i}^{T}$ and $\beta_{i}^{S}$. However, the final HBRB conclusion is jointly determined by the intermediate states and the top-level rule activations. Different combinations of intermediate states and rule activations may produce similar final outputs. Therefore, $p_{i}^{T}\approx \beta_{i}^{S}⇏I_{i}^{T}\approx I_{i}^{S},$ where $I_{i}^{T}$ and $I_{i}^{S}$ denote the internal diagnostic information of the teacher and student models, respectively. The preceding relation shows that matching the final outputs does not guarantee consistency in the internal diagnostic process. Output-only distillation may therefore produce an HBRB with a similar final decision but different intermediate states and rule activation paths.

**Problem** **3.** *Lack of quantitative evaluation for hierarchical inference. Suppose that the state belief distribution of the q-th sub-BRB is $s_{i,q}^{S}$, and the top-level rule activation distribution is $\omega_{i}^{S}$. The internal inference process of the HBRB can then be represented as $x_{i}\to {\left\{s_{i,q}^{S}\right\}}_{q=1}^{Q}\to \omega_{i}^{S}\to \beta_{i}^{S}.$*

Classification metrics such as accuracy, precision, and Macro-F1 evaluate only the final fault output. They do not indicate whether the intermediate-state distributions and rule activation distributions are reasonable. Two models may produce the same classification results while relying on different intermediate states and rule paths. Consequently, final classification metrics alone cannot determine whether the hierarchical reasoning process is stable or whether a diagnostic conclusion can be traced back through the intermediate states and activated rules. This constitutes the third problem addressed in this study.

## 3. Proposed HSD-BRB Method

This section is organized as follows. Section 3.1 presents the overall framework of HSD-BRB. Section 3.2 describes the expert-guided hierarchical semantic modeling method. Section 3.3 introduces the hierarchical knowledge distillation method, including knowledge transfer at the output, state, and path levels, together with the overall objective function and parameter constraints. Section 3.4 presents the traceable diagnosis and consistency evaluation procedures, including diagnostic-chain construction, state and path consistency, rule-semantic preservation, and path stability.

### 3.1. Overall Framework

**Figure 2:**
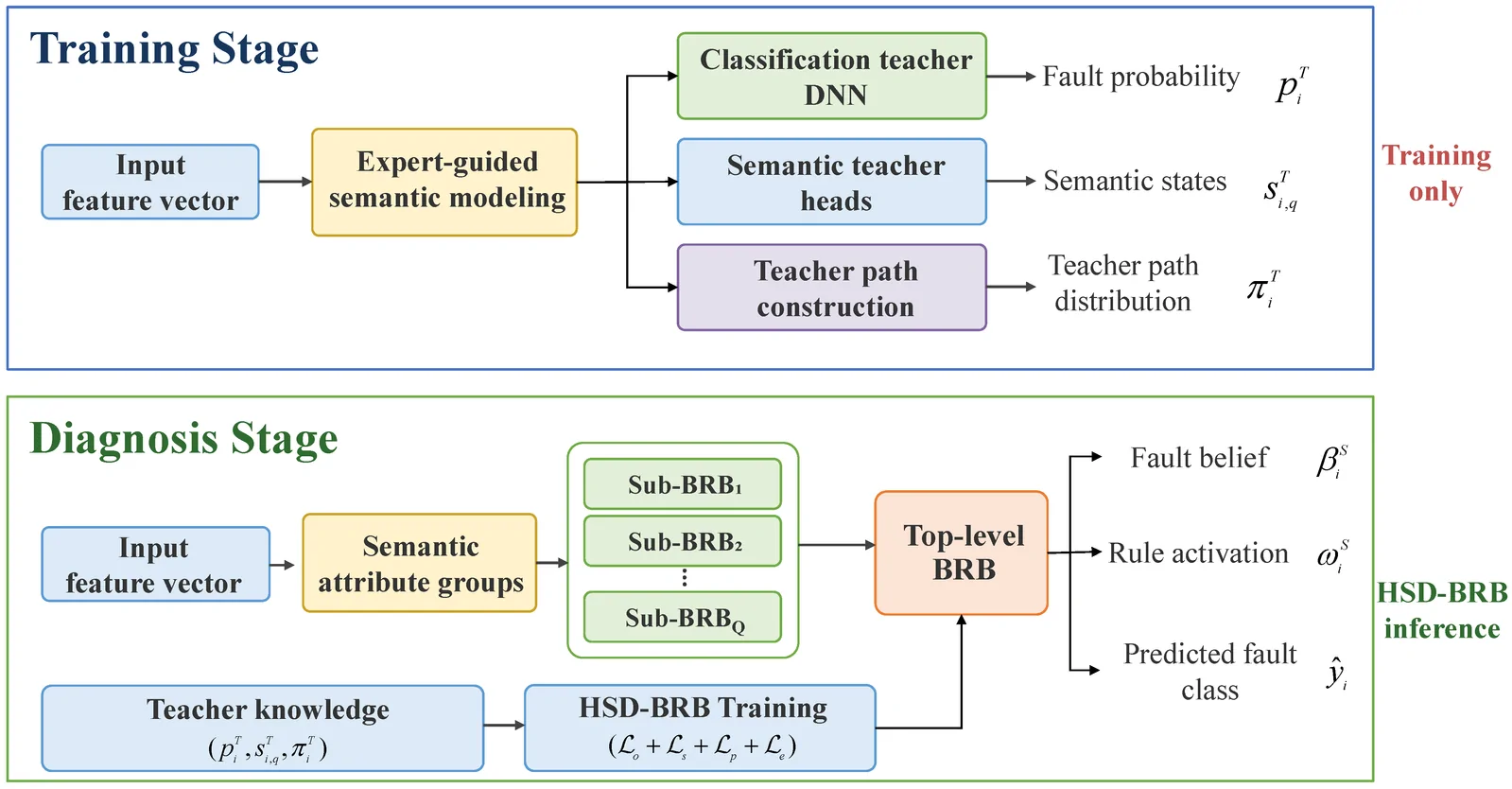
Overall framework of HSD-BRB.

The overall framework of HSD-BRB is shown in Figure 2. The framework consists of four closely related components: expert-guided semantic modeling, teacher-knowledge construction, hierarchical knowledge distillation, and HBRB-based diagnosis. First, the input attributes are organized into expert-defined semantic groups, and the corresponding sub-BRBs provide explicit intermediate states for hierarchical inference. During training, a classification teacher and auxiliary semantic teacher heads generate fault and semantic-state distributions from the complete input, which are used to supervise the corresponding levels of the HBRB. The resulting knowledge is transferred through output-, state-, and path-level distillation, together with an expert-deviation constraint that preserves the initial rule knowledge. After training, the teacher networks are removed, and the HBRB performs diagnosis independently through the intermediate semantic states, top-level rule activations, and final fault beliefs.

For the *i*-th input sample, let $x_{i}=\left[x_{i,1},x_{i,2},\ldots ,x_{i,M}\right].$ Based on domain knowledge concerning the monitored attributes, fault mechanisms, and their relationships, an attribute-grouping mapping G is defined in advance to organize the complete input into *Q* semantic attribute subsets: $G\left(x_{i}\right)=\left\{x_{i,1},x_{i,2},\ldots ,x_{i,Q}\right\}$. Here, $x_{i,q}$ denotes the attribute subset associated with the *q*-th semantic subspace. The attribute grouping is not learned automatically from the data; it is specified before model construction according to domain knowledge. Each semantic attribute subset is assigned to one sub-BRB to describe the corresponding intermediate state. The outputs of all sub-BRBs are then passed to the top-level BRB to obtain the final fault conclusion.

The teacher side consists of one classification teacher model and *Q* separately trained auxiliary semantic teacher heads. During training, all teacher-side networks access the complete input feature vector as privileged information, whereas each student sub-BRB receives only its designated semantic attribute subset. This training-only information asymmetry follows the generalized distillation setting [19]. At inference time, the teacher-side networks are removed and the hierarchical BRB operates independently. The classification teacher receives the complete input and produces the fault-class probability distribution: $p_{i}^{T}=f_{T}\left(x_{i}\right)=\left[p_{i,1}^{T},p_{i,2}^{T},\ldots ,p_{i,C}^{T}\right],$ where $f_{T}\left(\cdot \right)$ denotes the classification teacher model and $p_{i,c}^{T}$ is the probability that sample $x_{i}$ belongs to fault class *c*. The *q*-th auxiliary semantic teacher head also receives the complete input and produces the corresponding semantic-state distribution: $s_{i,q}^{T}=h_{q}^{T}\left(x_{i}\right)=\left[s_{i,q,1}^{T},s_{i,q,2}^{T},\ldots ,s_{i,q,H_{q}}^{T}\right],$ where $h_{q}^{T}\left(\cdot \right)$ denotes the *q*-th auxiliary semantic teacher head, and $H_{q}$ is the number of states in the corresponding semantic subspace. The auxiliary semantic teacher heads are supervised using state information defined from domain knowledge, so that their outputs share the same state space as the corresponding sub-BRBs. The classification teacher and auxiliary semantic teacher heads are trained separately and do not share network parameters.

The teacher path support is not directly produced by the classification teacher. Instead, it is constructed from the state distributions generated by the auxiliary semantic teacher heads. For a path $r_{l}=\left(h_{1},h_{2},\ldots ,h_{Q}\right),$ its unnormalized teacher path-support score is defined as

(5) $$\begin{array}{c} \pi_{i,l}^{T}=\prod_{q=1}^{Q}s_{i,q,h_{q}}^{T},\;l=1,\ldots ,L_{T}, \end{array}$$

where $h_{q}$ denotes the state selected by path $r_{l}$ in the *q*-th semantic subspace and $L_{T}=\prod_{q=1}^{Q}H_{q}$ is the total number of top-level paths. The product constructs path targets from the semantic-head outputs without training a separate joint-output model. Computing the full target still requires $L_{T}$ path scores. It is a factorized reference distribution conditioned on the input $x_{i}$, not an estimate of all cross-branch dependencies. $\pi_{i}^{T}=\left[\pi_{i,1}^{T},\pi_{i,2}^{T},\ldots ,\pi_{i,L_{T}}^{T}\right].$

On the student side, the *q*-th sub-BRB receives its corresponding semantic attribute subset and produces an intermediate-state belief distribution: $s_{i,q}^{S}={\mathrm{BRB}}_{q}\left(x_{i,q}\right),q=1,\ldots ,Q.$

The state belief distributions generated by all sub-BRBs are then provided as inputs to the top-level BRB. Through rule matching, rule activation, and evidential reasoning, the top-level BRB produces the top-level rule activation distribution and the final fault belief distribution:

(6) $$\begin{array}{c} \left(\omega_{i}^{S},\beta_{i}^{S}\right)={\mathrm{BRB}}_{T}\left(s_{i,1}^{S},s_{i,2}^{S},\ldots ,s_{i,Q}^{S}\right), \end{array}$$

Here, $\omega_{i}^{S}=\left[\omega_{i,1}^{S},\ldots ,\omega_{i,L_{T}}^{S}\right]$ denotes the top-level rule activation distribution, and $\beta_{i}^{S}=\left[\beta_{i,1}^{S},\ldots ,\beta_{i,C}^{S}\right]$ denotes the final fault belief distribution. The correspondence between the teacher knowledge and the HBRB reasoning structure is defined as $p_{i}^{T}↔\beta_{i}^{S}$, $s_{i,q}^{T}↔s_{i,q}^{S}$, $\pi_{i}^{T}↔\omega_{i}^{S}$.

The first pair represents the final fault output, the second pair corresponds to the semantic states of the sub-BRBs, and the third pair represents the top-level rule paths. These correspondences allow the teacher knowledge to be transferred to the fault-output, intermediate-state, and rule-path levels according to the hierarchy of the HBRB.

Training combines label supervision, output distillation, semantic-state distillation, path distillation, and an expert-deviation penalty. Their weighted objective is given in Equation (19); the individual terms are defined in Section 3.3.

The operation of HSD-BRB consists of a training stage and a diagnosis stage. During training, the classification teacher model and the auxiliary semantic teacher heads are first trained to obtain the teacher fault probabilities and semantic-state distributions. The teacher path distribution is then constructed, and the consequent belief degrees, rule weights, and attribute weights of the HBRB are optimized using the multilevel knowledge objectives. During diagnosis, the teacher model and auxiliary semantic teacher heads are no longer involved in inference. Only the trained HBRB is retained. The diagnostic process is expressed as $x_{i}\to {\left\{s_{i,q}^{S}\right\}}_{q=1}^{Q}\to \omega_{i}^{S}\to \beta_{i}^{S}\to {\hat{y}}_{i}$.

The predicted fault class is determined by ${\hat{y}}_{i}=\arg\max_{c\in \{1,\ldots ,C\}}\beta_{i,c}^{S}.$

Thus, HSD-BRB produces the fault class and its belief distribution while retaining the semantic-state distributions of the sub-BRBs and the activation distribution of the top-level rules. These intermediate results support subsequent explanation and consistency analysis of the diagnostic process. The expert-guided semantic modeling, hierarchical knowledge distillation, and diagnosis consistency evaluation are described in the following sections.

### 3.2. Expert-Guided Hierarchical Semantic Modeling

Expert knowledge defines the attribute groups, intermediate-state meanings, and initial rule relationships. This initialization provides a structured starting point for learning. The expert-deviation penalty then discourages changes to the initial consequent beliefs, helping retain these relationships during optimization [8,13].

The expert initialization was formulated by the authors using the physical interpretation of the extracted features, the fault characteristics represented in the bearing and gearbox datasets, and the established BRB construction procedure. The Energy, Impact, and Spectrum groupings, the Low/Medium/High semantic states, and the qualitative fault prototypes were specified from these domain considerations. The numerical reference values were calibrated from the training data, whereas the initial belief degrees and rule weights were calculated using the initialization equations in Appendix B. Table A1, Table A2, Table A3 and Table A4 therefore report reproducible expert-guided initializations rather than independently elicited probability assessments from a separate expert panel.

For attribute *m* in group *q*, the ordered reference values are $A_{q,m}=\left\{A_{q,m,1},\ldots ,A_{q,m,J_{q,m}}\right\}$, with $A_{q,m,1}<\ldots <A_{q,m,J_{q,m}}$. Their numerical locations are calibrated from training data only.

Each sub-BRB rule corresponds to a combination of attribute reference states. A predefined mapping $\Psi_{q}$ assigns nonnegative expert scores to the possible intermediate states; normalization converts these scores into a complete consequent belief distribution. A corresponding mapping $\Psi_{T}$ initializes the top-level fault beliefs from semantic-state combinations. Rule and attribute weights complete the initial parameter set $\Theta^{0}$. The calibration, normalization, and weight-initialization formulas are retained in Appendix B.

The construction therefore proceeds from attribute grouping and reference-value calibration to sub-BRB initialization and then top-level initialization. The rule count follows Equation (4). Different groups may have different numbers of attributes, reference values, and intermediate states. This structure supplies the initial semantic states and rule paths used in the distillation objectives below.

### 3.3. Hierarchical Knowledge Distillation

**Figure 3:**
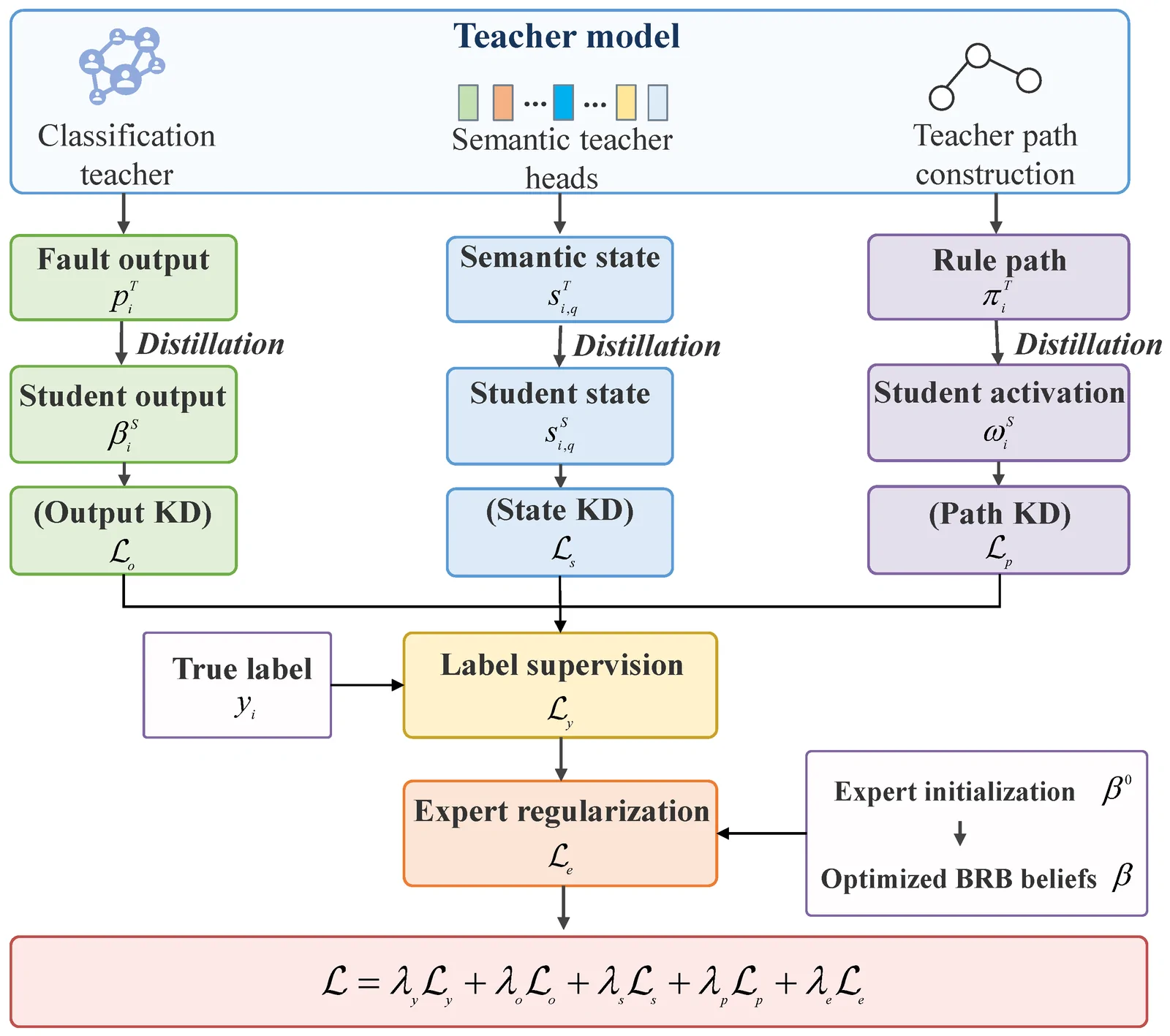
Hierarchical knowledge distillation and loss construction in HSD-BRB.

Output-level distillation alone does not ensure agreement in intermediate inference (Problem 2, Section 2.2). HSD-BRB therefore aligns fault outputs, semantic states, and rule paths with the corresponding student beliefs and activations, as shown in Figure 3 [14,15,16]. The teacher networks remain fixed while the student’s consequent beliefs, rule weights, and attribute weights are optimized.

#### 3.3.1. Output-Level Knowledge Transfer

Let the ground-truth label of sample $x_{i}$ be $y_{i}$, with one-hot encoding $y_{i}=\left[y_{i,1},y_{i,2},\ldots ,y_{i,C}\right]$. If a sample belongs to fault class *c*, then $y_{\left\{i,c\right\}}=1$; otherwise, $y_{\left\{i,c\right\}}=0$. According to the HSD-BRB output definition, the fault belief distribution produced by HSD-BRB is $\beta_{i}^{S}$. The label-supervision loss is defined as

(7) $$\begin{array}{c} L_{y}=-\frac{1}{N}\sum_{i=1}^{N}\sum_{c=1}^{C}y_{i,c}\log\left(\beta_{i,c}^{S}+\varepsilon \right), \end{array}$$

where ε>0 is introduced for numerical stability.

The classification teacher produces fault probabilities, whereas HSD-BRB produces fault beliefs. To establish a soft correspondence between the two, a temperature parameter *T* is first used to smooth an arbitrary distribution *v* [14]:

(8) $$\begin{array}{c} {\mathrm{Soft}}_{T}{\left(v\right)}_{c}=\frac{v_{c}^{1/T}}{\sum_{r=1}^{C}v_{r}^{1/T}}. \end{array}$$

Because the current HBRB uses complete belief distributions and does not explicitly include an unknown state, the smoothed teacher probabilities are combined with a uniform distribution to form the teacher soft-belief target:

(9) $$\begin{array}{c} {\tilde{p}}_{i}^{T}=\left(1-\eta \right){\mathrm{Soft}}_{T}\left(p_{i}^{T}\right)+\frac{\eta }{C}1, \end{array}$$

where η∈[0,1] is the uncertainty-mixing coefficient and 1 is a C-dimensional all-one vector. This operation retains the class preference of the teacher while reducing the influence of excessively concentrated probabilities on the BRB belief distribution.

The student fault belief distribution is smoothed using the same temperature:

(10) $$\begin{array}{c} {\tilde{\beta }}_{i}^{S}={\mathrm{Soft}}_{T}\left(\beta_{i}^{S}\right). \end{array}$$

The output distillation loss is defined as

(11) $$\begin{array}{c} L_{o}=\frac{T^{2}}{N}\sum_{i=1}^{N}D_{\mathrm{KL}}\left({\tilde{p}}_{i}^{T}\|{\tilde{\beta }}_{i}^{S}\right), \end{array}$$

where the Kullback–Leibler divergence [20] is defined as

(12) $$\begin{array}{c} D_{\mathrm{KL}}\left(a\|b\right)=\sum_{j}a_{j}\log\frac{a_{j}}{b_{j}}. \end{array}$$

The factor $T^{2}$ compensates for the change in loss scale introduced by temperature smoothing. The label-supervision loss encourages the model to identify the correct fault class, whereas the output distillation loss enables the top-level BRB to learn the relative class-discrimination information contained in the teacher model [14].

#### 3.3.2. State- and Path-Level Knowledge Transfer

State-level distillation encourages the sub-BRB belief distributions to match the auxiliary semantic teacher outputs, addressing semantic ambiguity (Problem 1, Section 2.2). Path-level distillation encourages agreement between top-level rule activations and the path distribution constructed from those outputs. Together, these objectives guide intermediate inference in addition to supervising the final prediction [15].

Let the state distributions produced by the *q*-th semantic teacher head and the corresponding sub-BRB be denoted by $s_{i,q}^{T}$ and $s_{i,q}^{S}$, respectively. The semantic-state distillation loss is defined as

(13) $$\begin{array}{c} L_{s}=\frac{1}{NQ}\sum_{i=1}^{N}\sum_{q=1}^{Q}D_{\mathrm{KL}}\left(s_{i,q}^{T}\|s_{i,q}^{S}\right). \end{array}$$

Expanding the KL divergence gives

(14) $$\begin{array}{c} L_{s}=\frac{1}{NQ}\sum_{i=1}^{N}\sum_{q=1}^{Q}\sum_{h=1}^{H_{q}}s_{i,q,h}^{T}\log\frac{s_{i,q,h}^{T}}{s_{i,q,h}^{S}}. \end{array}$$

This loss directly constrains the outputs of the sub-BRBs, allowing them to learn the intermediate semantic information provided by the auxiliary semantic teacher heads rather than adjusting these states only indirectly through the final fault labels.

Path-level supervision uses the factorized reference distribution defined in Equation (5). The teacher and student path scores are normalized before their divergence is evaluated.

The teacher path distribution is obtained by combining the state distributions generated by the auxiliary semantic teacher heads. Based on the teacher path definition, the path-support values are normalized as follows:

(15) $$\begin{array}{c} {\bar{\pi }}_{i,l}^{T}=\frac{\pi_{i,l}^{T}}{\sum_{r=1}^{L_{T}}\pi_{i,r}^{T}},\;l=1,\ldots ,L_{T}. \end{array}$$

Interpreted probabilistically, the product imposes conditional independence of the semantic states given $x_{i}$ within the reference model. This is a modeling assumption, not a claim that the measured feature groups are physically independent. Section 4.4.3 describes their observed associations; those correlations do not test conditional independence given the full input.

The student path distribution is obtained from the activation weights of the top-level rules:

(16) $$\begin{array}{c} {\bar{\omega }}_{i,l}^{S}=\frac{\omega_{i,l}^{S}}{\sum_{r=1}^{L_{T}}\omega_{i,r}^{S}}. \end{array}$$

Since the top-level rule activation weights are generally normalized, the preceding equation mainly ensures that the teacher and student path distributions have the same mathematical form.

The path distillation loss between the normalized teacher path distribution and the normalized student rule-activation distribution is defined as

(17) $$\begin{array}{c} L_{p}=\frac{1}{N}\sum_{i=1}^{N}D_{\mathrm{KL}}\left({\bar{\pi }}_{i}^{T}\|{\bar{\omega }}_{i}^{S}\right). \end{array}$$

State distillation constrains each sub-BRB to preserve the local semantic states, whereas path distillation constrains the combination and activation of multiple semantic states in the top-level rules. These two losses act on the intermediate-state and rule-path levels of the HBRB, respectively, allowing the student model to retain hierarchical diagnostic information while learning the final fault decision [15,16].

#### 3.3.3. Overall Objective and Parameter Constraints

An expert-deviation penalty discourages changes to the initialized consequent beliefs during optimization [13]. Let $B_{q}$ and $B_{q}^{0}$ denote the current and initial consequent-belief matrices of sub-BRB *q*, and let $B_{T}$ and $B_{T}^{0}$ denote those of the top-level BRB. The penalty is

(18) $$\begin{array}{c} L_{e}=\frac{1}{Q+1}\left[\sum_{q=1}^{Q}\frac{{∥B_{q}-B_{q}^{0}∥}_{F}^{2}}{L_{q}H_{q}}+\frac{{∥B_{T}-B_{T}^{0}∥}_{F}^{2}}{L_{T}C}\right]. \end{array}$$

This loss measures only the deviation of the consequent belief distributions from their expert initialization. It does not constrain attribute reference values, attribute grouping, rule weights, or attribute weights.

The complete objective balances classification through $L_{y}$ and $L_{o}$, state and path agreement through $L_{s}$ and $L_{p}$, and expert preservation through $L_{e}$:

(19) $$\begin{array}{c} L=\lambda_{y}L_{y}+\lambda_{o}L_{o}+\lambda_{s}L_{s}+\lambda_{p}L_{p}+\lambda_{e}L_{e}, \end{array}$$

Here, $\lambda_{y},\lambda_{o},\lambda_{s},\lambda_{p},\lambda_{e}\geq 0$ control the contributions of the corresponding supervision and regularization terms. Different weight settings can be used to obtain output-focused, state-focused, path-focused, or expert-preserving training configurations. The hyperparameter sensitivity analysis in Section 4.7 examines the effects of these weight choices on diagnostic performance and reasoning consistency.

The learnable parameter set of HSD-BRB is defined as

(20) $$\begin{array}{c} \Theta ={\left\{B_{q},\theta_{q},\delta_{q}\right\}}_{q=1}^{Q}\cup \left\{B_{T},\theta_{T},\delta_{T}\right\}, \end{array}$$

where *B*, θ, and δ denote the consequent belief degrees, rule weights, and attribute weights, respectively.

Softmax and sigmoid transformations maintain valid BRB parameters during optimization. The consequent beliefs are parameterized as

(21) $$\begin{array}{c} \beta_{q,h,k}=\frac{\exp(z_{q,h,k})}{\sum_{r=1}^{H_{q}}\exp\left(z_{q,r,k}\right)}. \end{array}$$

The rule weights and attribute weights are parameterized using sigmoid functions:

(22) $$\begin{array}{c} \theta_{q,k}=\frac{1}{1+\exp(-a_{q,k})},\;\delta_{q,m}=\frac{1}{1+\exp(-d_{q,m})}. \end{array}$$

The expert initialization is performed in the constrained parameter space. Because a finite sigmoid logit cannot represent the boundary value 1 exactly, parameters initialized at 1 are clipped to $1-10^{-4}$ when packed into the unconstrained optimization vector by logitClip; the displayed expert attribute weights remain 1. The rule weights listed in Appendix A are the effective initialized values before this packing step. The top-level BRB uses the same parameterization. Consequently,

(23) $$\begin{array}{c} \sum_{h=1}^{H_{q}}\beta_{q,h,k}=1,\;0<\theta_{q,k}<1,\;0<\delta_{q,m}<1. \end{array}$$

The final model parameters are obtained by minimizing the overall objective function:

(24) $$\begin{array}{c} \Theta^{*}=\arg\min_{\Theta }L\left(\Theta \right). \end{array}$$

During optimization, the classification teacher and auxiliary semantic heads remain fixed and supply the output, state, and path targets [14,21]. After training, the teacher-side models are removed, and the HBRB independently produces fault beliefs, intermediate semantic states, and top-level rule activations.

### 3.4. Traceable Diagnosis and Consistency Evaluation

HSD-BRB provides a diagnostic chain through named semantic states and activated belief rules. Conventional black-box classifiers can provide class probabilities and post hoc feature attributions, but do not generally expose this hierarchical inference structure directly. The following consistency metrics address Problem 3 (Section 2.2) by evaluating intermediate states and rule activations alongside classification performance [8].

HSD-BRB performs fault classification while retaining the semantic-state belief distributions of the sub-BRBs, the activation distribution of the top-level rules, and the final fault belief distribution. These intermediate inference results form a diagnostic chain consisting of the input evidence, semantic states, rule paths, and final fault conclusion. The consistency between the student model and the teacher knowledge can then be evaluated at both the state and path levels.

#### 3.4.1. Diagnosis Path Construction

For sample $x_{i}$, the diagnostic process follows the diagnostic-chain definition. To identify the dominant evidence at each reasoning level, the dominant state of the *q*-th semantic sub-BRB is defined as

(25) $$\begin{array}{c} {\hat{h}}_{i,q}=\arg\max_{h\in \{1,\ldots ,H_{q}\}}s_{i,q,h}^{S},\;q=1,\ldots ,Q,\; \end{array}$$

where $s_{i,q,h}^{S}$ is the belief degree assigned by the *q*-th semantic sub-BRB to its *h*-th state. The resulting dominant semantic-state combination is

(26) $$\begin{array}{c} {\hat{r}}_{i}=\left({\hat{h}}_{i,1},{\hat{h}}_{i,2},\ldots ,{\hat{h}}_{i,Q}\right).\; \end{array}$$

The top-level rule with the largest activation weight is selected as the dominant diagnostic path:

(27) $$\begin{array}{c} {\hat{l}}_{i}=\arg\max_{l\in \{1,\ldots ,L_{T}\}}\omega_{i,l}^{S}.\; \end{array}$$

Because a diagnostic conclusion may be supported by multiple rules, the single most activated rule may not fully represent the reasoning basis. Therefore, a cumulative rule set is selected by accumulating rule activations until a predefined threshold τ is reached:

(28) $$\begin{array}{c} R_{i}^{\tau }=\underset{R\subseteq \{1,\ldots ,L_{T}\}}{\arg\min}\;\left|R\right|,\;s.t.\;\sum_{l\in R}\omega_{i,l}^{S}\geq \tau ,\; \end{array}$$

where the rules are sorted in descending order of activation and τ∈(0,1] is the cumulative activation threshold.

For the predicted class ${\hat{y}}_{i}$, the support of the *l*-th top-level rule is defined as

(29) $$\begin{array}{c} \rho_{i,l}=\frac{\omega_{i,l}^{S}\beta_{{\hat{y}}_{i},l}^{T}}{\sum_{j=1}^{L_{T}}\omega_{i,j}^{S}\beta_{{\hat{y}}_{i},j}^{T}+\varepsilon }. \end{array}$$

Here, $\beta_{{\hat{y}}_{i},l}^{T}$ is the consequent belief assigned to the predicted class by top-level BRB rule *l*, with the class index preceding the rule index as in Appendix B. The superscript *T* denotes the top level, not the teacher. The small positive constant ε avoids division by zero. The value $\rho_{i,l}$ describes the rule’s relative support for the predicted class, not a causal contribution.

Accordingly, the diagnostic chain for sample $x_{i}$ is represented as

(30) $$\begin{array}{c} E_{i}=\left\{x_{i},{\left\{s_{i,q}^{S}\right\}}_{q=1}^{Q},\omega_{i}^{S},R_{i}^{\tau },\beta_{i}^{S},{\hat{y}}_{i}\right\}.\; \end{array}$$

This chain records the input evidence, semantic states, activated rules, and final fault conclusion in sequence, allowing the diagnostic result to be traced through the hierarchical reasoning structure of the HBRB.

#### 3.4.2. Diagnostic State and Path Consistency

State consistency and path consistency quantify the alignment between the student BRB’s internal reasoning and the teacher model’s intermediate representations. These metrics address Problem 2 (Section 2.2) by evaluating whether similar final outputs are produced through similar intermediate states and rule activations, rather than through different reasoning paths that happen to converge to the same conclusion [15,16].

State consistency measures how closely the semantic-state distributions learned by the sub-BRBs match those produced by the auxiliary semantic teacher heads. For the test set $D_{\mathrm{te}}$, state consistency is evaluated using the mean KL divergence:

(31) $$\begin{array}{c} D_{\mathrm{state}}=\frac{1}{|D_{\mathrm{te}}|Q}\sum_{i\in D_{\mathrm{te}}}\sum_{q=1}^{Q}\sum_{h=1}^{H_{q}}\;{\tilde{s}}_{i,q,h}^{T}\log\frac{\;{\tilde{s}}_{i,q,h}^{T}}{\;{\tilde{s}}_{i,q,h}^{S}},\; \end{array}$$

Here, ${\tilde{s}}_{i,q}^{T}$ and ${\tilde{s}}_{i,q}^{S}$ denote the teacher and student state distributions after smoothing and normalization, respectively:

(32) $$\begin{array}{c} {\tilde{s}}_{i,q,h}=\frac{s_{i,q,h}+\varepsilon }{\sum_{j=1}^{H_{q}}\left(s_{i,q,j}+\varepsilon \right)}.\; \end{array}$$

Path consistency evaluates the agreement between the activation process of the top-level rules and the path knowledge constructed from the semantic teacher states. It is defined as

(33) $$\begin{array}{c} D_{\mathrm{path}}=\frac{1}{|D_{\mathrm{te}}|}\sum_{i\in D_{\mathrm{te}}}\sum_{l=1}^{L_{T}}\;{\tilde{\pi }}_{i,l}^{T}\log\frac{{\tilde{\pi }}_{i,l}^{T}}{\;{\tilde{\omega }}_{i,l}^{S}},\; \end{array}$$

Here, ${\tilde{\pi }}_{i}^{T}$ and ${\tilde{\omega }}_{i}^{S}$ denote the normalized teacher path distribution and student top-level rule activation distribution, respectively.

Lower $D_{\mathrm{state}}$ and $D_{\mathrm{path}}$ indicate closer agreement with the teacher’s semantic-state targets and constructed path targets, respectively. They assess two complementary aspects of knowledge transfer: the intermediate states identified and the rule combinations activated. These measures complement classification metrics; a lower divergence alone does not imply a correct fault prediction.

#### 3.4.3. Rule-Semantic Preservation and Path Stability

To evaluate the relationship between the optimized rules and the initial expert knowledge, the consequent belief distribution of rule *k* is first converted into an expected state:

(34) $$\begin{array}{c} u_{q,k}=\sum_{h=1}^{H_{q}}v_{q,h}\beta_{q,h,k},\; \end{array}$$

where $\beta_{q,h,k}$ denotes the belief degree assigned by rule *k* of sub-BRB *q* to the *h*-th ordered state and $v_{q,h}$ is the numerical score assigned to that state. In the experiments, the ordered states Low, Medium, and High are assigned scores 1, 2, and 3, respectively. For two rules $\left(k,k^{\prime }\right)$ that are adjacent only in the *m*-th antecedent state, a positive expert direction is preserved when $u_{q,k^{\prime }}\geq u_{q,k}$; for a negative direction, the criterion is $u_{q,k^{\prime }}\leq u_{q,k}$. The set P below contains adjacent rule pairs together with their predefined direction.

For an adjacent rule pair $\left(k,k^{\prime }\right)$, let $d_{k,k^{\prime }}=+1$ denote a positive expert direction and $d_{k,k^{\prime }}=-1$ denote a negative direction. The direction indicator is defined as

(35) $$\begin{array}{c} I_{\mathrm{dir}}\left(k,k^{\prime }\right)=\left\{\begin{array}{cc} I(u_{q,k^{\prime }}\geq u_{q,k}), & d_{k,k^{\prime }}=+1,\\ I(u_{q,k^{\prime }}\leq u_{q,k}), & d_{k,k^{\prime }}=-1. \end{array}\right. \end{array}$$

The direction-preservation rate over all adjacent rule pairs is then defined as

(36) $$\begin{array}{c} R_{\mathrm{dir}}=\frac{1}{\left|P\right|}\sum_{(k,k^{\prime })\in P}I_{\mathrm{dir}}\left(k,k^{\prime }\right). \end{array}$$

Here, P denotes the set of adjacent rule pairs with a specified ordering relationship. A larger $R_{\mathrm{dir}}$ indicates that the optimized rule base better preserves the state transition directions specified by the expert.

To further compare the dominant diagnostic paths of the optimized model and the expert-initialized HBRB on the same samples, the expert path preservation rate is defined as

(37) $$\begin{array}{c} R_{\mathrm{path}}=\frac{1}{|D_{\mathrm{te}}|}\sum_{i\in D_{\mathrm{te}}}I\left({\hat{l}}_{i}^{S}={\hat{l}}_{i}^{E}\right),\; \end{array}$$

where ${\hat{l}}_{i}^{S}$ and ${\hat{l}}_{i}^{E}$ denote the dominant top-level rule paths of the optimized HSD-BRB and the expert-initialized HBRB, respectively. This metric reflects whether data optimization changes the dominant diagnostic path. Strictly speaking, the expert-path equation measures expert-path preservation rather than the robustness of the model to input perturbations.

For repeated independent training runs, path stability is defined as the agreement rate of the dominant paths obtained for the same test samples:

(38) $$\begin{array}{c} R_{\mathrm{run}}=\frac{2}{K(K-1)}\sum_{a=1}^{K-1}\sum_{b=a+1}^{K}\frac{1}{|D_{\mathrm{te}}|}\sum_{i\in D_{\mathrm{te}}}I\left({\hat{l}}_{i}^{\left(a\right)}={\hat{l}}_{i}^{\left(b\right)}\right),\; \end{array}$$

where *K* denotes the number of independent runs, and ${\hat{l}}_{i}^{\left(a\right)}$ represents the dominant path generated for test sample *i* in the *a*-th run. A higher $R_{\mathrm{run}}$ indicates that the model produces more stable diagnostic paths under different parameter initializations and optimization processes.

By jointly considering the final fault belief distribution, state consistency, path consistency, rule-direction preservation, and path stability, HSD-BRB can be evaluated from three levels: the final diagnostic result, the intermediate semantic states, and the rule-based reasoning process. These metrics reflect the traceability of the model’s internal inference and its consistency with teacher and expert knowledge. They should not, however, be interpreted directly as evidence of causal explanations.

## 4. Case Study

Section 4.1 defines the diagnosis problem, and Section 4.2 describes model construction. Section 4.3 evaluates bearing diagnosis through performance comparisons, consistency analysis, and diagnostic explanations. Section 4.4 extends the evaluation to gearboxes, including semantic teacher comparisons, paired significance tests, semantic correlations, and feature-noise robustness. Section 4.5 and Section 4.6 examine raw-vibration noise and out-of-range feature perturbations, respectively. Section 4.7 analyzes hyperparameter sensitivity, and Section 4.8 reports computational cost and deployment constraints. Section 4.9 and Section 4.10 summarize the findings and discuss practical limitations.

Table 2 summarizes the main experimental configurations.

### 4.1. Problem Formulation

Rotating machinery is widely used in energy, manufacturing, transportation, and aerospace systems. As key transmission components, bearings and gearboxes directly affect transmission stability, operational safety, and maintenance costs. Timely identification of abnormal conditions from vibration monitoring signals can help prevent fault propagation, reduce the risk of unplanned downtime, and support condition-based maintenance [22].

Fault diagnosis of rotating machinery generally involves inferring the current operating condition and corresponding fault type from vibration signals. In practice, the measured response is jointly affected by structural transmission, load variation, fault severity, and measurement noise. As a result, different faults may produce similar signal patterns, while the same fault may show substantial feature variation across samples.

This case study focuses on three issues arising from complex vibration responses: distinguishing between similar fault conditions, establishing the relationship between fault evidence and diagnostic conclusions, and supporting engineering verification of the results. Different faults may simultaneously cause changes in vibration energy, increased transient impacts, and spectral redistribution. The resulting coupling among fault features makes similar conditions difficult to distinguish accurately. In addition to identifying the final fault class, an engineering diagnostic model should indicate which vibration evidence corresponds to each equipment state and how these states jointly support the conclusion. When different faults exhibit similar vibration responses, the model should also provide traceable evidence so that engineers can inspect the main signal features, intermediate state assessments, and rule-based reasoning process, and verify whether the resulting diagnosis is consistent with the actual operating mechanism of the equipment.

### 4.2. Construction of HSD-BRB

The fault diagnosis performance of HSD-BRB is first evaluated using the Southeast University (SEU) bearing dataset. The dataset was collected using a drivetrain dynamic simulator [6]. It contains five operating states: healthy condition, rolling-element fault, inner-race fault, outer-race fault, and compound inner–outer race fault, covering multiple combinations of rotational speed and load. The bearing experiment uses the dataset condition labelled 20 Hz–0 V. Here, 20 Hz denotes the nominal speed-frequency setting of the test rig, and 0 V denotes its load-voltage setting. These are operating-condition settings, not vibration amplitudes. The voltage specifies the loading setting rather than a mechanical torque in N·m. The original condition labels are retained because an independently measured shaft speed and a voltage-to-torque calibration have not been verified for the recordings used in this study.

**Figure 4:**
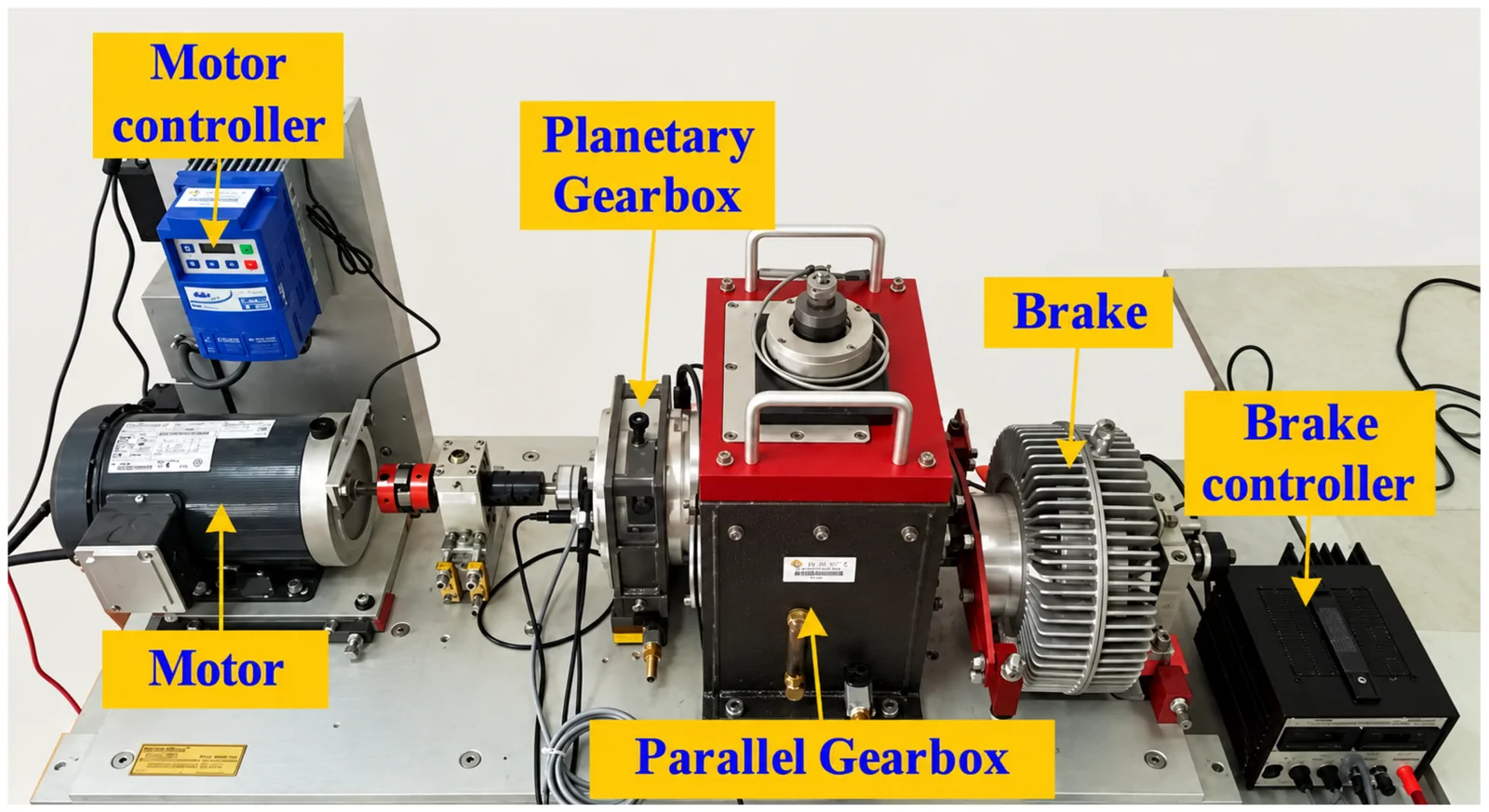
Experimental drivetrain rig associated with the Southeast University bearing and gearbox datasets. The labelled components include the motor, motor controller, planetary gearbox, parallel gearbox, brake, and brake controller.

Figure 4 shows the experimental drivetrain rig used to acquire the vibration data. The motor provides the rotating input, the planetary and parallel gearboxes form the transmission path, and the brake and its controller impose the loading condition. The figure is included to clarify the physical arrangement of the test platform; the numerical operating-condition labels used in the experiments are retained from the dataset documentation.

The second vibration channel in the original data is used. The continuous vibration signal is divided into independent segments of 4096 sampling points, and six time- and frequency-domain features are extracted [23,24] from each segment. The five fault states and their label encoding are listed in Table 3.

**Figure 5:**
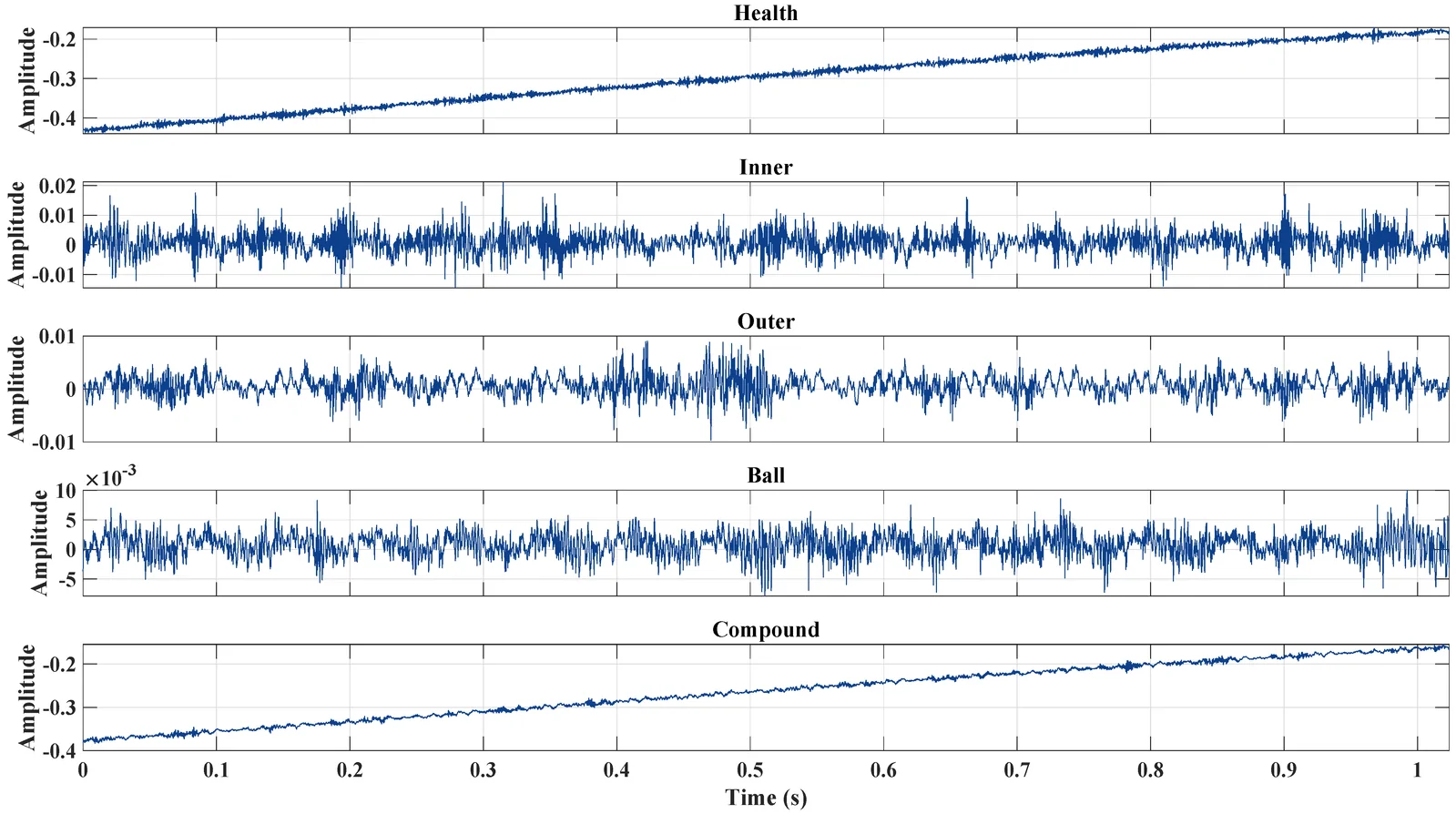
Representative time-domain vibration signals for healthy and faulty bearing conditions under the 20 Hz–0 V setting.

Figure 5 presents representative time-domain signals from the five bearing conditions. The signals are shown in the original amplitude units provided by the dataset, and the displayed segment spans approximately 1.024 s at the 4000 Hz sampling frequency. The figure illustrates differences in amplitude and transient structure between the conditions; it is used as a qualitative view of the measured data, whereas the reported diagnosis results are based on the six features extracted from each 4096-point segment.

The data are divided into training and test sets using a 70–30% split. The feature-normalization parameters, attribute reference values, and teacher models are determined using the training set only and then fixed for the test set to prevent data leakage.

Based on the effects of bearing faults on overall vibration energy, transient impacts, and spectral distribution, the six input attributes are organized into three semantically meaningful subspaces, as summarized in Table 4.

The inputs to the three sub-BRBs are defined as

(39) $$\begin{array}{c} x_{i,E}=\left[x_{i,1},x_{i,2}\right],\;x_{i,I}=\left[x_{i,3},x_{i,4}\right],\;x_{i,S}=\left[x_{i,5},x_{i,6}\right].\; \end{array}$$

Each sub-BRB produces a three-state belief distribution:

(40) $$\begin{array}{c} s_{i,q}^{S}=\left[s_{i,q,1}^{S},s_{i,q,2}^{S},s_{i,q,3}^{S}\right],\;q\in \left\{E,I,S\right\}. \end{array}$$

For each input attribute, three ordered reference values are defined as Low (L), Medium (M), and High (H), corresponding to the 10th, 50th, and 90th percentiles of the standardized training data, respectively. The gearbox dataset follows the same reference-value construction principle, but its reference values are calculated independently from the gearbox training set and do not use the bearing-data reference values.

Since each sub-BRB contains two input attributes and each attribute has three reference values, each sub-BRB contains 3 × 3 = 9 rules. The three sub-BRBs therefore contain 27 lower-level rules in total. Each sub-BRB produces three intermediate states, so the top-level BRB also contains 3 × 3 × 3 = 27 rules.

The initial consequent belief distributions of the three sub-BRBs are provided in Table A2, Table A3 and Table A4, respectively, while the initial consequent belief distribution of the top-level BRB is given in Table A1. All initial belief distributions are specified according to the bearing fault mechanisms and are subsequently calibrated during training through label supervision and hierarchical knowledge distillation.

A rule in the top-level BRB can be expressed as follows:

(41) $$\begin{array}{cc} \; & R_{l}^{T}:\mathrm{If}\;E=e_{l}\wedge I=i_{l}\wedge S=s_{l}, \end{array}$$

(42) $$\begin{array}{cc} \; & \;\mathrm{Then}\;\{\beta_{\mathrm{Health},l},\beta_{\mathrm{Inner},l},\beta_{\mathrm{Outer},l},\beta_{\mathrm{Ball},l},\beta_{\mathrm{Compound},l}\}. \end{array}$$

For example, the antecedent of Rule 27 in Table A1 is (H,H,H), indicating high Energy, strong Impact, and a high composite Spectrum state. This rule has the highest initial support for the compound fault, while retaining nonzero belief in the other fault classes to reflect overlapping vibration responses. To obtain fault knowledge with stronger discriminative capability, a deep teacher model is trained using the six-dimensional feature vector. Its input is $x_{i}=\left[x_{i,1},x_{i,2},\ldots ,x_{i,6}\right],$ and its output is the probability distribution over the five bearing conditions:

(43) $$\begin{array}{c} p_{i}^{T}=f_{T}\left(x_{i}\right)=\left[p_{i,1}^{T},p_{i,2}^{T},\ldots ,p_{i,5}^{T}\right], \end{array}$$

Here, $p_{i,c}^{T}$ denotes the probability that sample *i* belongs to class *c*. The teacher model is implemented as a multilayer fully connected neural network. It receives the six normalized features, processes them through several fully connected layers with nonlinear activations, and produces the five fault probabilities through a softmax output layer:

(44) $$\begin{array}{c} \sum_{c=1}^{5}p_{i,c}^{T}=1,\;p_{i,c}^{T}\geq 0.\; \end{array}$$

The classification teacher is trained using the cross-entropy loss:

(45) $$\begin{array}{c} L_{T}=-\frac{1}{N}\sum_{i=1}^{N}\sum_{c=1}^{5}I\left(y_{i}=c\right)\log p_{i,c}^{T}.\; \end{array}$$

In addition to the classification teacher, three independent auxiliary semantic teacher heads are constructed for the Energy, Impact, and Spectrum subspaces. Each auxiliary semantic teacher head receives the complete six-dimensional input and produces a three-state probability distribution:

(46) $$\begin{array}{c} s_{i,q}^{T}=h_{q}^{T}\left(x_{i}\right)=\left[s_{i,q,L}^{T},s_{i,q,M}^{T},s_{i,q,H}^{T}\right],\;q\in \left\{E,I,S\right\},\; \end{array}$$

where L, M, and H denote the low, medium, and high semantic states, respectively. Each semantic teacher head satisfies

(47) $$\begin{array}{c} \sum_{r\in \{L,M,H\}}s_{i,q,r}^{T}=1.\; \end{array}$$

The supervision targets of the auxiliary semantic teacher heads are generated by mapping the corresponding attribute groups to the ordered Low, Medium, and High intervals defined by the training-set reference values. The target states use the same three-state space as the consequent states of the corresponding sub-BRB. Specifically, the Energy head learns the energy state, the Impact head learns the impact state, and the Spectrum head learns the spectral state.

The classification teacher and the three auxiliary semantic teacher heads are trained separately and do not share network parameters. After training, the classification teacher produces $p_{i}^{T}$, while the three semantic heads produce $s_{i,E}^{T}$, $s_{i,I}^{T}$, and $s_{i,S}^{T}$. These three semantic-state distributions are further used to construct the top-level rule-path knowledge:

(48) $$\begin{array}{c} \pi_{i,l}^{T}=s_{i,E,e_{l}}^{T}s_{i,I,i_{l}}^{T}s_{i,S,s_{l}}^{T},\;l=1,\ldots ,27.\; \end{array}$$

where $\left(e_{l},i_{l},s_{l}\right)$ denotes the Energy, Impact, and Spectrum state combination associated with the *l*-th top-level rule. Accordingly, the teacher model provides three types of distillation knowledge:

(49) $$\begin{array}{cc} \; & p_{i}^{T}\to \mathrm{fault}\text{-}\mathrm{output}\;\mathrm{knowledge}, \end{array}$$

(50) $$\begin{array}{cc} \; & \{s_{i,E}^{T},s_{i,I}^{T},s_{i,S}^{T}\}\to \mathrm{semantic}\text{-}\mathrm{state}\;\mathrm{knowledge}, \end{array}$$

(51) $$\begin{array}{cc} \; & \pi_{i}^{T}\to \mathrm{rule}\text{-}\mathrm{path}\;\mathrm{knowledge}. \end{array}$$

The teacher models are used only to generate soft supervision during training. Once HSD-BRB has been optimized, the teacher models no longer participate in fault inference on the test set. All test samples are diagnosed solely by the trained hierarchical BRB. For the bearing dataset, the classification teacher achieves a test Accuracy of 97.92% and a Macro-F1 of 0.9791, while the gearbox teacher achieves 99.48% Accuracy and a Macro-F1 of 0.9948. The gearbox teacher is trained using the complete original training partition, whereas the BRB is optimized using a fixed stratified subset of approximately 10% of the training samples; the corresponding settings are described in Section 4.4. For the bearing dataset, the Energy, Impact, and Spectrum semantic teacher heads achieve test Accuracies of 91.17%, 95.84%, and 92.73%, respectively. These results describe the teacher-side supervision used during distillation rather than the final performance of HSD-BRB.

### 4.3. Fault Diagnosis Based on HSD-BRB

#### 4.3.1. Diagnostic Performance

To evaluate the bearing fault diagnosis performance of HSD-BRB, each configuration was trained independently three times. The ablation configurations are defined independently for the bearing and gearbox experiments according to the respective experimental settings. The mean and standard deviation of Accuracy and Macro-F1 are reported in Table 5.

The ablation results show a clear progression in the role of each training signal. Adding output and semantic-state distillation to the label-supervised baseline increases Accuracy from 88.05% (A1) to 95.58% (A2), a joint gain of 7.53 percentage points. Path distillation gives a further increase to 96.28% in A3. Adding the expert-deviation constraint reduces Accuracy slightly to 95.76% in A4, but retains a high diagnostic level while restricting changes to the initial expert rule structure. Thus, the constraint contributes primarily to the structure and inspectability of the learned BRB, rather than to Accuracy alone. The independently trained six-feature teacher reaches 97.9221% Accuracy and 0.9791 Macro-F1 and is used only to generate training targets.

**Figure 6:**
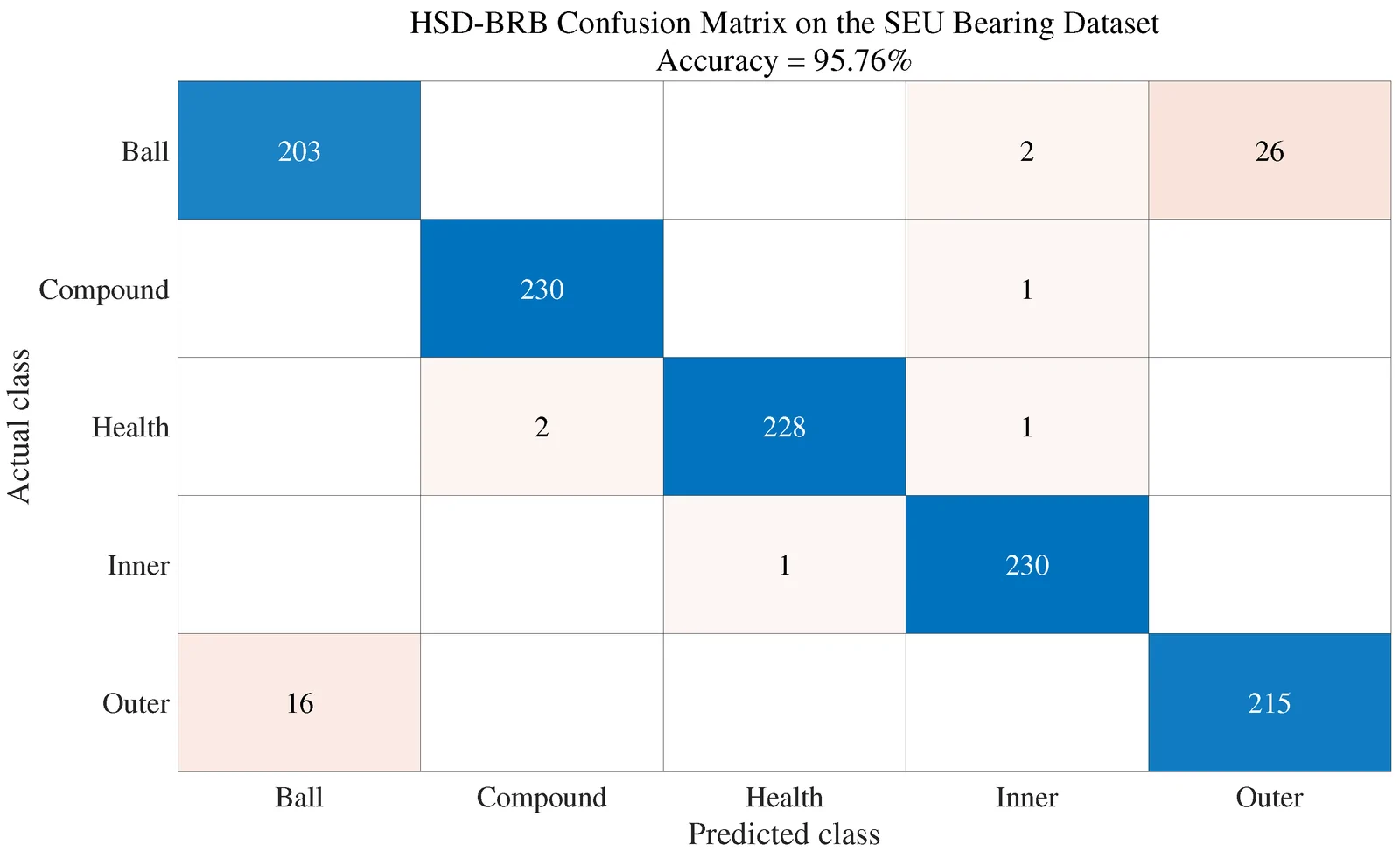
Confusion matrix of HSD-BRB on the SEU bearing dataset.

Figure 6 presents the confusion matrix of HSD-BRB on the Southeast University bearing dataset. Most samples are located on the diagonal, indicating strong overall classification performance. The main errors occur between the Outer and Ball classes, which is consistent with the similar impact and spectral responses produced by these two local fault types. The result agrees with the Accuracy and Macro-F1 values reported in Table 5. A plausible explanation for this error pattern is that outer-race and ball faults can generate overlapping impulsive components and similar spectral energy distributions under the selected operating condition. The available feature groups may therefore provide limited separation between these two responses.

Classification scores alone do not describe the semantic states or rule activations behind each decision. The following analyses compare HSD-BRB with external baselines and then examine its state and path consistency.

#### 4.3.2. Comparison with External Baselines

To complement the internal ablation study, HSD-BRB is compared with an RBF-SVM, a random forest, a one-versus-rest ANFIS classifier, and a fixed-weight flat BRB on the bearing dataset. All methods use the same six input features and the original fixed training and test partitions. Baseline settings are specified before evaluation, without selecting hyperparameters according to test performance. The six-feature DNN teacher is included as a reference. Ten matched evaluations are used for the HSD-BRB and external-baseline significance analysis; the fixed-weight Flat-BRB and ANFIS outputs are deterministic across these evaluations.

The RBF-SVM uses one-versus-one classification, a box constraint of 1, training-based standardization, and an automatically estimated kernel scale. The random forest contains 300 trees, with two candidate features per split and a minimum leaf size of 1. ANFIS uses five one-versus-rest zero-order Sugeno systems, each with two Gaussian membership functions per input and 100 training epochs. The flat BRB uses three training-set quantile references per feature, giving 729 rules. Its consequent beliefs are estimated from activation-weighted class counts with prior smoothing, while rule and attribute weights are fixed at one; predictions are obtained by evidential-reasoning aggregation. This flat BRB is used as a fixed-weight reference implementation rather than a fully optimized BRB.

Table 6 reports the separate matched evaluations used for the external-baseline comparison. The HSD-BRB obtains a mean Accuracy of 96.0520%, which differs slightly from the 95.76% mean reported for A4 in the main ablation study because the two results are based on different matched evaluation runs. In this external comparison, HSD-BRB improves mean Accuracy over the fixed-weight Flat-BRB by approximately 3.84 percentage points, and the paired test confirms this difference. RBF-SVM, ANFIS-OVR, and Random Forest obtain higher classification scores. HSD-BRB instead provides a specific form of diagnostic trace through semantic-state beliefs and hierarchical rule activations, examined in the following case analysis. The comparison therefore shows that the main advantage of HSD-BRB in this experiment lies in its structured diagnostic representation rather than the highest classification accuracy among the tested baselines.

Table 7 shows that all eight external comparisons remain significant after Holm correction. HSD-BRB is significantly better than the fixed-weight Flat-BRB and significantly worse than RBF-SVM, random forest, and ANFIS-OVR in both metrics. The fixed-weight Flat-BRB and ANFIS-OVR each have one unique fitted model repeated across the matched evaluations, so their zero standard deviations describe deterministic repeated outputs rather than training variability.

A separate structural comparison allows for both the hierarchical and flat BRBs to optimize their consequent beliefs, rule weights, and attribute weights. Both use the original 890 bearing training samples and 385 test samples, the same teacher output targets, and a common objective consisting of label supervision, output distillation (weight 0.5), and expert deviation (weight 0.02). State and path losses are omitted from both models because the flat BRB has no intermediate semantic nodes. The flat model’s initial consequent beliefs are obtained from the initialized hierarchical model at the 729 reference-grid points. Both fits use an initialization perturbation scale of 0.05, with SQP limits of 200 iterations and 50,000 function evaluations.

In Table 8, the hierarchical BRB achieves 1.0390 percentage points higher Accuracy and 0.0117 higher Macro-F1, with 47.2% less measured training time. Its smaller parameter space permits 178 SQP iterations within the specified budget, compared with 11 for the flat BRB. This comparison illustrates the optimization cost of the larger flat rule base under a common function-evaluation budget.

#### 4.3.3. Consistency Analysis

To examine whether HSD-BRB preserves the teacher model’s intermediate diagnostic knowledge, state-level and rule-path consistency are compared across the different configurations. State-KL measures the difference between the semantic-state belief distributions produced by the sub-BRBs and those produced by the auxiliary semantic teacher heads. Path-KL measures the difference between the top-level rule activation distribution and the path distribution constructed from the teacher model. Lower values indicate higher consistency between the student and teacher at the corresponding reasoning level.

**Figure 7:**
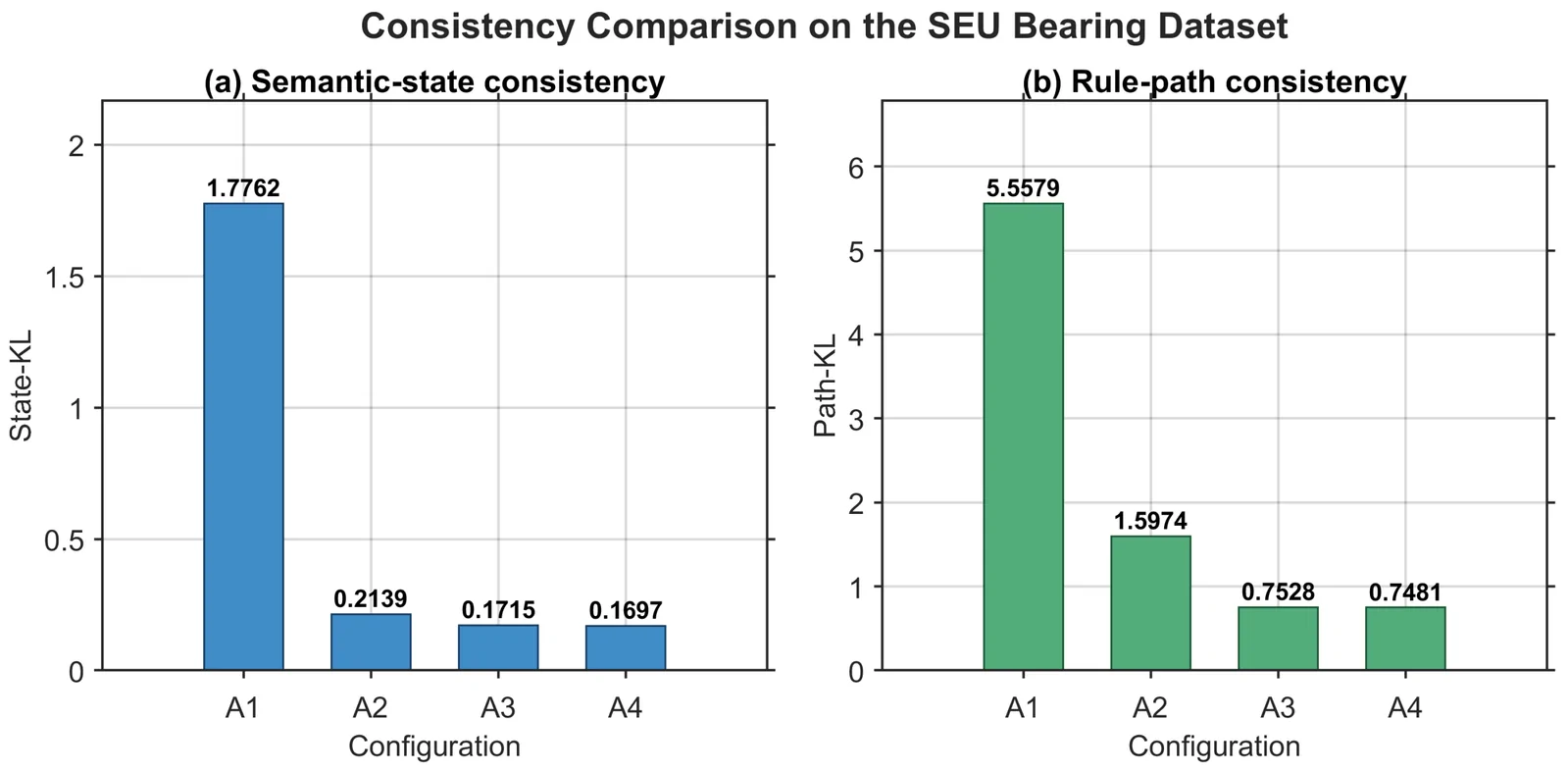
Consistency comparison of different HSD-BRB configurations on the SEU bearing dataset: (**a**) State-KL; (**b**) Path-KL.

The results in Table 9 and Figure 7 follow the intended hierarchy of the distillation objectives. Label supervision alone gives high State-KL and Path-KL. Adding output and semantic-state distillation reduces State-KL from 1.7762 to 0.2139, while path distillation reduces Path-KL from 1.5974 to 0.7528. The expert-deviation constraint produces a further, smaller reduction, giving A4 the lowest State-KL (0.1697) and Path-KL (0.7481). The added path objective primarily improves rule-activation agreement, while the expert constraint limits changes to the initialized consequent beliefs.

#### 4.3.4. Case-Level Diagnostic Analysis

To illustrate the diagnostic chain of HSD-BRB and its ability to integrate information from multiple semantic sources, two representative samples are selected from the bearing test set, as shown in Table 10. The dominant states are (M,M,M) for Case 1 and (H,L,H) for Case 2. These labels refer to different feature groups, so matching or differing labels do not by themselves establish agreement or conflict about the fault class. Both samples are correctly classified by the complete HSD-BRB.

In Case 1, each sub-BRB assigns its highest belief to its own M state, producing the combination (M,M,M). This combination mainly activates top-level rule $R_{14}$, and rule aggregation assigns a final belief of 0.9876 to the Inner class. The example traces the decision through the three group-specific states and the activated rule without treating the M labels as identical physical evidence.

In Case 2, the Energy and Spectrum branches assign beliefs of 0.8847 and 0.8159 to H, respectively, while the Impact branch assigns 0.9995 to L. These operational states are defined relative to training-set references rather than physical fault thresholds. In particular, Spectrum combines spectral centroid and spectral bandwidth, so its H state cannot be interpreted as high spectral spread alone. The L state of the Impact branch represents a low-impact state under the kurtosis/crest-factor mapping, but is not sufficient on its own to establish health.

The combination (H,L,H) mainly activates $R_{21}$, with an activation of 0.9542. The trained top-level BRB aggregates the activated rules and produces a final Health belief of 0.9945. Thus, the Health prediction reflects the learned mapping from the combined branch beliefs to fault classes, rather than a premise that high Energy and Spectrum states imply health. This case illustrates the model’s inference under the tested data distribution, not a general physical rule for other operating conditions. Neither the activation of $R_{21}$ nor the final Health belief alone establishes how frequently this state combination occurs among healthy training samples. The diagnostic chains for the two cases are

(52) $$\begin{array}{cc} x_{78} & \to \left(M,M,M\right)\to R_{14}\to \mathrm{Inner},\\ x_{1} & \to \left(H,L,H\right)\to R_{21}\to \mathrm{Health}. \end{array}$$

These examples demonstrate how the final fault decision can be traced from the input evidence through semantic states and activated rules to the diagnostic conclusion.

#### 4.3.5. Comparison with Post Hoc Explanations

Post hoc explanations were evaluated for the teacher and the complete bearing HSD-BRB configuration (A4) on 25 test samples, with five samples selected from each class. Interventional Shapley attributions (SHAP) [25] were computed by enumerating all 64 coalitions of the six features, using a fixed background of 50 training samples with ten samples per class. The computation is exact for this empirical background. Each explanation targeted the corresponding model’s predicted class; the models predicted different classes for one of the 25 cases. The attribution summaries therefore describe each model’s own decisions rather than a uniformly matched-class comparison.

Table 11 shows that the teacher’s attribution is concentrated on spectral centroid, whereas variance, spectral centroid, and RMS have the largest mean absolute attributions for HSD-BRB. These patterns describe contributions to model scores under the selected background distribution, not physical causality or explanation correctness. The interventional feature replacements can also produce combinations that do not preserve observed dependencies among vibration features. A more distributed attribution pattern alone does not establish better interpretability.

LIME-style [26] continuous weighted linear surrogates were fitted using 1000 Gaussian perturbations per case and repeated five times. Perturbation standard deviations were set to half the training-feature standard deviations. The distance kernel used training-standardized distances with width $\sqrt{6}$, and the ridge coefficient was $10^{-3}$. Both models used matched perturbations. This implementation is a LIME-style surrogate rather than the discretized public LIME implementation. Weighted $R^{2}$ and MAE were calculated on the same perturbations used to fit each surrogate and therefore measure in-sample local fit, not held-out explanation fidelity.

As shown in Table 12, HSD-BRB achieves a slightly higher mean weighted $R^{2}$ than the teacher, whereas the teacher obtains lower weighted MAE and slightly lower coefficient variability. Overall, the two models show comparable local surrogate fidelity, with no clear advantage in post hoc surrogate performance. Beyond feature-level attribution, HSD-BRB provides intrinsic diagnostic information through its intermediate semantic-state beliefs, activated rules, and diagnostic paths. As illustrated in Table 10, these quantities provide a direct and traceable view of how the final fault decision is formed.

### 4.4. Cross-Component Validation on the Gearbox Dataset

To evaluate the applicability of HSD-BRB to different rotating machinery components, additional experiments are conducted on the Southeast University (SEU) gearbox dataset. The dataset contains five operating conditions: Health, Chipped, Miss, Root, and Surface. Their label encoding is listed in Table 13.

The gearbox experiment uses the dataset condition labelled 30 Hz–2 V, corresponding to a nominal speed-frequency setting of 30 Hz and a load-voltage setting of 2 V. One vibration channel is selected from the original measurements. The fixed dataset partition contains 890 training samples and 385 held-out test samples, with 178 training and 77 test samples per fault class. For the small-sample HSD-BRB experiment, a stratified subset of 90 training samples (18 per class, approximately 10.1% of the original training partition) is used to optimize the BRB configurations. The remaining training samples are excluded from BRB optimization, while the held-out test set remains unchanged.

The gearbox experiments use the six-dimensional feature representation and the three semantic sub-BRB structures defined in Section 4.2, with the input attributes organized into the Energy, Impact, and Spectrum semantic spaces. The reference values and normalization parameters are determined independently from the gearbox training data. The classification teacher is trained using the complete 890-sample training partition with an internal fit/validation split, whereas each HSD-BRB configuration is optimized using the fixed stratified subset of 90 samples. Thus, the teacher provides privileged supervision during training, while both teacher and student are evaluated on the same held-out test partition of 385 samples, which is not used for training or model selection.

**Figure 8:**
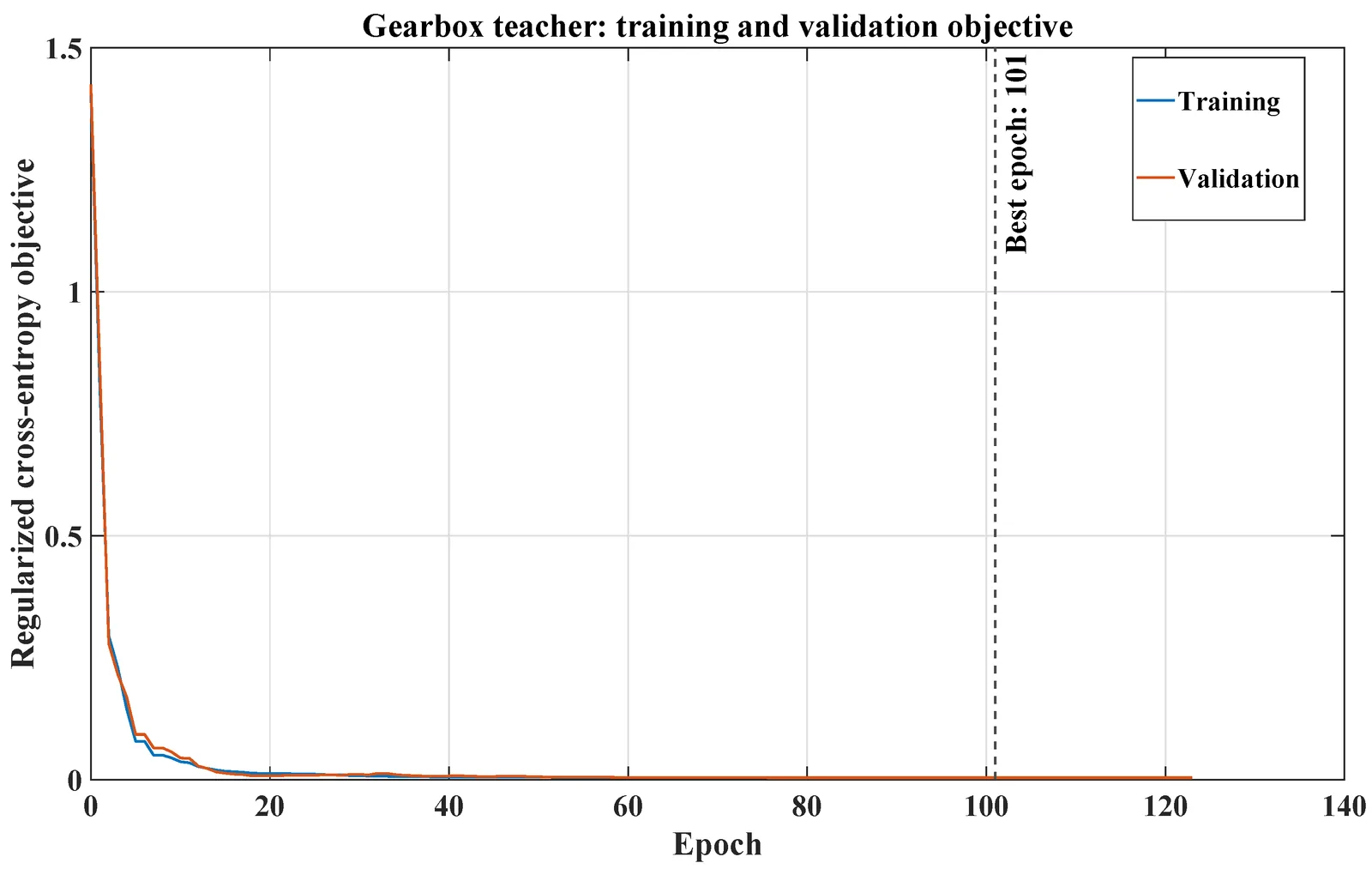
Training and validation regularized cross-entropy objectives of the gearbox classification teacher. The best validation epoch is marked by the vertical dashed line.

The gearbox classification teacher is a fully connected network with layer widths 6–64–32–5 and 2693 trainable parameters. It is trained on the complete original training partition using an 85%/15% stratified fit/validation split, while the held-out test set is kept entirely separate. Early stopping is applied based on the validation objective. The teacher achieves 99.89% Accuracy and 0.9989 Macro-F1 on the complete training partition, 100.00% Accuracy and 1.0000 Macro-F1 on the validation partition, and 99.48% Accuracy and 0.9948 Macro-F1 on the held-out test partition. The training and validation curves in Figure 8 show similar trends, with no clear indication of substantial overfitting. The teacher uses the complete training partition and provides privileged supervision during BRB training.

To assess whether classification distillation depends on additional labelled teacher data, classification teachers with the same 6–64–32–5 architecture were trained using either the fixed 90-sample student subset or the complete 890-sample training partition. Both teachers used internal validation and early stopping. All students used the same 90 labelled samples and 385 test samples, with matched initialization and a common SQP budget of 200 iterations and 50,000 objective-function evaluations. Three repetitions compared label supervision with the expert constraint, output distillation, and complete distillation. In the complete-distillation configurations, the semantic teachers were also fitted using the corresponding 90 or 890 samples. Normalization, reference values, and expert initialization followed the original experimental settings; the control therefore restricts teacher fitting data while retaining shared preprocessing. Table 14 reports the results.

The 90-sample classification teacher achieves a mean Accuracy of 98.7013%, compared with 99.0476% for the 890-sample teacher. Among the student configurations in Table 14, output distillation from the 90-sample teacher yields the highest mean Accuracy, 97.2294%, exceeding the label-supervised reference by 0.2597 percentage points. Complete distillation from the same teacher reaches 96.9697%, with a smaller standard deviation than the reference. The corresponding students using the 890-sample teachers achieve 96.8831% and 96.7965%, respectively. Under the shared preprocessing settings, a teacher fitted on the same labelled samples as the student can therefore provide useful output supervision, as reflected in the higher mean student Accuracy.

The expert rule matrix for the gearbox is also constructed independently rather than directly copied from the bearing experiment. Gear chipping and missing-tooth faults are mainly associated with increased meshing impacts, root faults reflect changes in structural responses, and surface faults are commonly accompanied by contact impacts and spectral spreading. The gearbox experiment therefore evaluates whether HSD-BRB remains applicable when both the fault types and the expert semantic relationships change.

#### 4.4.1. Effect of Semantic Teacher Design

An additional single-run analysis compares three semantic supervision sources on the gearbox data. Unlike the main 90-sample BRB ablation below, this exploratory comparison optimizes each student on the complete 890-sample training partition; its results must therefore not be interpreted as part of the small-label experiment.

Table 15 compares three sources of semantic supervision: a full-input MLP, a quantile-based mapping, and a subgroup-only MLP. In the single-run comparison, the full-input MLP and quantile mapping lead to the same student Accuracy and Macro-F1, while the subgroup-only MLP gives slightly lower values. The full-input MLP also provides lower State-KL and Path-KL, indicating closer alignment between the student and the corresponding teacher targets. Quantile mapping achieves 100% state-label accuracy by construction, since its outputs are directly determined by the quantile-based labeling rule. The full-input semantic heads are used only during training, allowing the teacher to incorporate information from all six features when generating targets for the individual sub-BRBs.

To examine the effect of state distillation, Table 16 compares the three semantic teachers with $\lambda_{s}=0$ and $\lambda_{s}=0.2$ while keeping $\lambda_{p}=0$. The remaining settings are unchanged, and each comparison uses three matched initialization runs.

Table 16 shows a consistent reduction in State-KL after enabling state distillation for all three semantic teachers. For the full-input MLP, State-KL decreases from 0.3255 to 0.2013; for quantile mapping, it decreases from 0.4375 to 0.2554; and for the subgroup-only MLP, it decreases from 0.3309 to 0.2073. The reduction is therefore observed both with full-input semantic supervision and with the more restrictive subgroup-only teacher. The effect on classification performance differs across teacher designs. With the full-input MLP and quantile mapping, Accuracy and Macro-F1 decrease slightly after state distillation, whereas the subgroup-only MLP improves from 96.5370% to 96.7100% in Accuracy and from 0.9651 to 0.9670 in Macro-F1. These results show that state distillation consistently improves agreement at the semantic-state level across the three teacher constructions, while its effect on final fault classification depends on the source of semantic supervision.

Table 17 summarizes the diagnostic performance and reasoning-consistency results on the gearbox dataset. Each configuration is run independently ten times, and the results are reported as the mean ± standard deviation.

The ablation results separate classification performance from agreement with the teacher’s internal outputs. Label supervision raises Accuracy from 92.18% in A0 to 95.53% in A1. Output and state distillation reduce State-KL from 0.5839 in A1 to 0.2656 in A3, with little change in Accuracy. Adding path distillation in A4 lowers Path-KL from 2.1024 to 1.1093, although Accuracy falls to 93.45%. The expert constraint in A5 raises Accuracy to 96.81% and Macro-F1 to 0.9680, the highest mean scores among the BRB configurations, while retaining low State-KL (0.2091). Its Path-KL (1.2486) is slightly higher than A4’s. The teacher reaches 99.48% Accuracy on the held-out test set. It was trained on the full 890-sample training partition, whereas the BRB student used 90 labelled samples; this is privileged teacher supervision.

**Figure 9:**
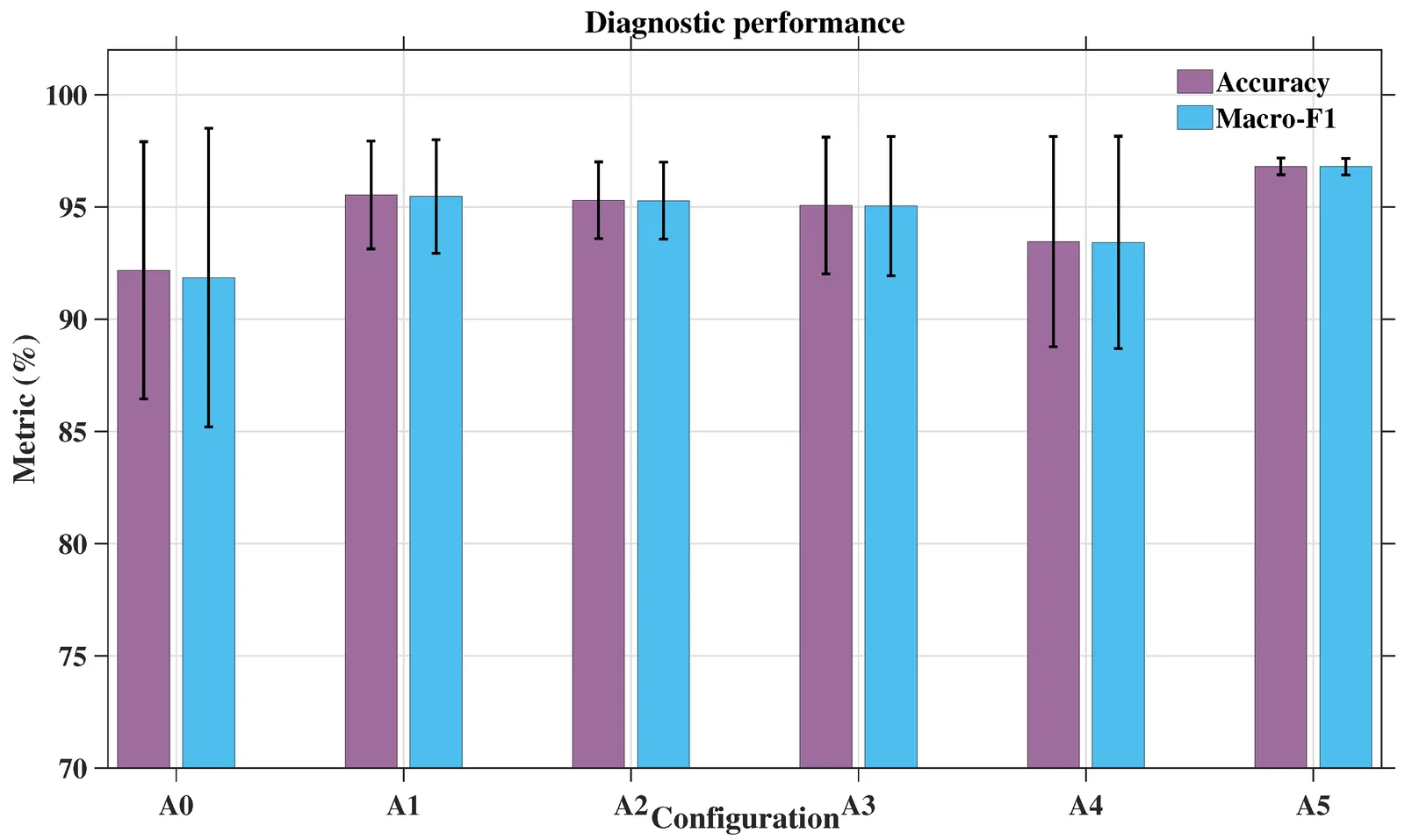
Diagnostic performance on the gearbox dataset.

**Figure 10:**
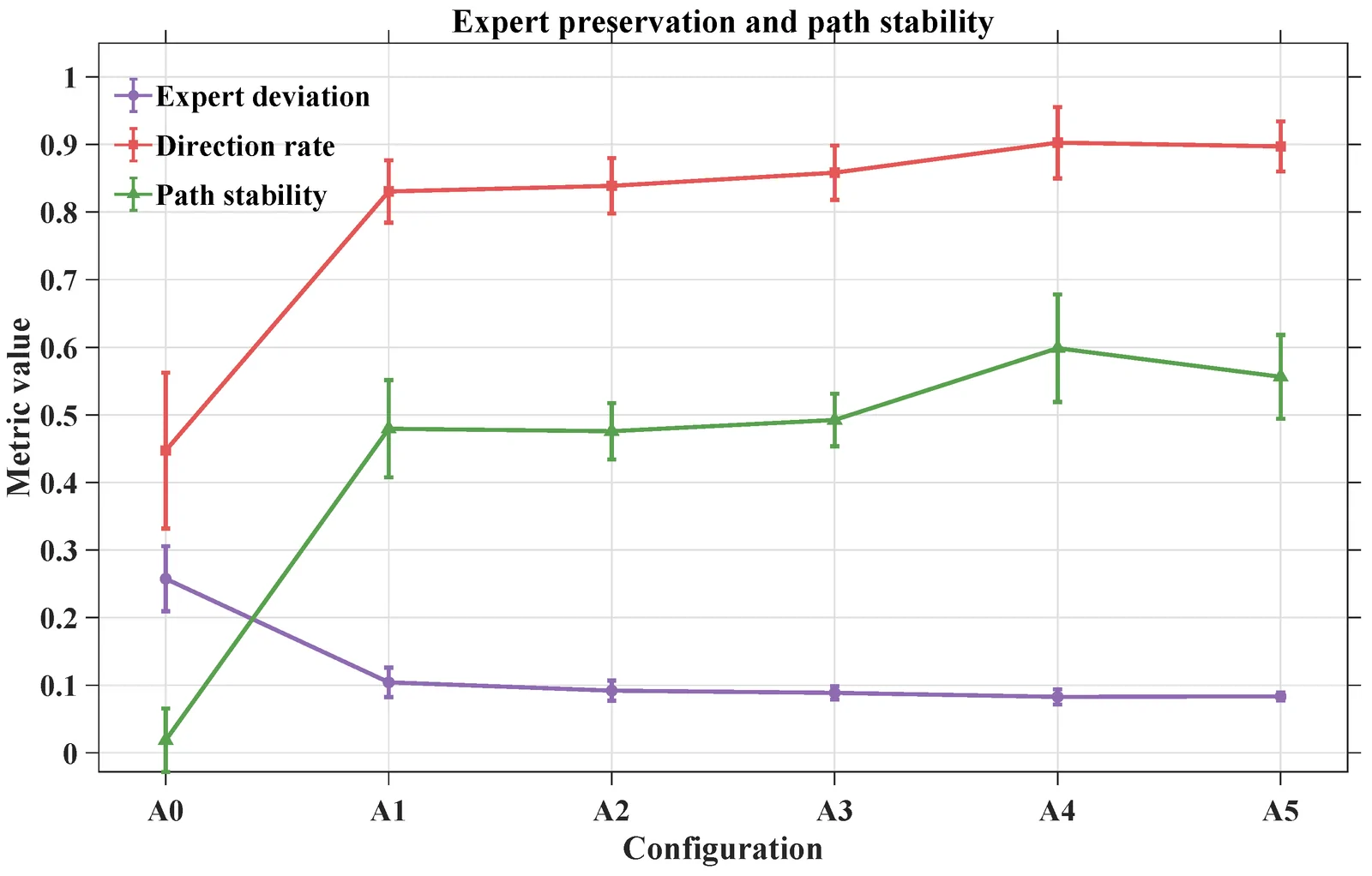
Expert preservation and path stability.

Figure 9 presents the diagnostic performance of the different configurations together with the corresponding run-to-run errors. Figure 10 presents the expert-knowledge preservation and diagnostic-path stability metrics. The error bars represent the standard deviations over ten independent runs.

Figure 9 shows the classification results and variation across ten runs. Figure 10 shows that Expert Deviation falls from approximately 0.26 in A0 to 0.08 in A5, while Rule Direction Rate rises from approximately 0.45 to 0.90. Path Stability increases from nearly zero to approximately 0.56. A5 has the smallest expert deviation and the highest rule-direction preservation rate; A4 retains a slight advantage in Path Stability. Table 17 reports the highest mean classification score for A5 in the main experiment, together with lower State-KL and better preservation of the initialized rules. This mean-score ranking alone does not establish a classification advantage, which is assessed separately by the paired tests in Table 18.

#### 4.4.2. Statistical Significance of Ablation Results

A5 was compared with A1, A3, and A4 using ten matched optimization starts on the same stratified gearbox subset. Each pair shared the test partition, solver budget, and teacher outputs. Accuracy and Macro-F1 were assessed with two-sided paired *t*-tests and exact Wilcoxon signed-rank tests; Holm correction was applied to the six comparisons.

Table 18 shows no significant difference after Holm correction. A5 exceeds A3 by 2.0260 percentage points in mean Accuracy, but its confidence interval includes zero. Its mean difference from A4 is 0.2338 points, and it is slightly below A1 in these matched runs. Thus, the higher mean scores in the main ablation are not accompanied by a statistically confirmed classification advantage in this supplementary paired experiment.

All 40 fits reached the common function-evaluation budget before convergence was confirmed (SQP exit flag 0). The paired results describe this fixed-budget protocol.

#### 4.4.3. Correlation Analysis of Semantic Branches

Because Energy, Impact, and Spectrum attributes may be physically coupled, the dependency structure underlying the factorized path construction was examined on both datasets. For each semantic branch, an expected state score was computed as

(53) $$z_{q}=s_{q,L}+2s_{q,M}+3s_{q,H},\;q\in \left\{E,I,S\right\}.$$

Spearman correlations were calculated separately for the original training and held-out test partitions. For the gearbox dataset, the original deterministic 10% student-training subset was also analyzed. The partitions were never pooled, and the semantic teacher outputs were not retrained for this analysis.

As shown in Table 19, the test-set correlations for the bearing dataset are −0.3531, 0.1420, and 0.2964 for the Energy–Impact, Energy–Spectrum, and Impact–Spectrum pairs, respectively. The corresponding gearbox values are 0.0002, −0.8553, and 0.2077. The strong negative Energy–Spectrum association in the gearbox data is also observed in the original training partition (−0.8793) and in the 10% student-training subset (−0.8860). Class-wise analysis further shows that global correlations can conceal class-dependent dependencies.

The feature-level analysis gives a consistent result. On the bearing test set, the Spearman correlation between RMS and spectral centroid is 0.8254, whereas the corresponding value on the gearbox test set is −0.8490. These results show associations among the measured features and semantic scores across samples; they do not test conditional independence of semantic states given the complete input. Accordingly, Equation (5) should be interpreted as a factorized reference distribution for path distillation rather than as an exact joint posterior. The construction remains computationally tractable and preserves the hierarchical decomposition, but it does not explicitly model cross-branch dependencies.

To quantify the practical impact of the factorized path approximation in Equation (5), a single matched comparison replaced the product-based target with a joint estimate from a 6–32–16–27 auxiliary network. The network was trained on the original training partition using joint labels formed from the three mapping-defined semantic states, with internal validation. Both students used the same 90 training samples, 385 test samples, initial parameters, and solver budget; all other supervision targets and loss weights were unchanged. Table 20 reports this one-run matched comparison separately from the ten-run main experiment.

Within the additional matched comparison, replacing the factorized target with the joint target raises Accuracy from 87.7922% to 89.3506% and Macro-F1 from 0.8769 to 0.8924, while reducing State-KL from 0.2254 to 0.2050. These values are used only to compare the two path-target constructions under the same initialization and fixed solver budget; they do not replace or directly compare with the ten-run main result of 96.8100%. The experiment shows that HSD-BRB can accommodate a directly learned joint target, while the factorized construction retains the practical benefit of generating path supervision directly from the semantic heads.

#### 4.4.4. Feature-Noise Robustness

To evaluate the sensitivity of the model to input perturbations, Gaussian noise with different intensities is added to the six-dimensional standardized features of the gearbox test set. The perturbed feature is defined as

(54) $$\begin{array}{c} {\tilde{x}}_{i,m}=x_{i,m}+\alpha \epsilon_{i,m}\sigma_{m}^{\mathrm{tr}},\;\epsilon_{i,m}∼N\left(0,1\right),\; \end{array}$$

where $\sigma_{m}^{\mathrm{tr}}$ is the standard deviation of the *m*-th feature in the training set, and α denotes the noise level. The experiments use α∈{0,0.05,0.10,0.20,0.30}. The relative degradation in Accuracy and Macro-F1 is calculated as

(55) $$\begin{array}{c} D_{\mathrm{Acc}}\left(\alpha \right)=\frac{\mathrm{Acc}(0)-\mathrm{Acc}(\alpha )}{\mathrm{Acc}(0)}, \end{array}$$

(56) $$\begin{array}{c} D_{F1}\left(\alpha \right)=\frac{\mathrm{Macro}\text{-}F1(0)-\mathrm{Macro}\text{-}F1(\alpha )}{\mathrm{Macro}\text{-}F1(0)} \end{array}$$

**Figure 11:**
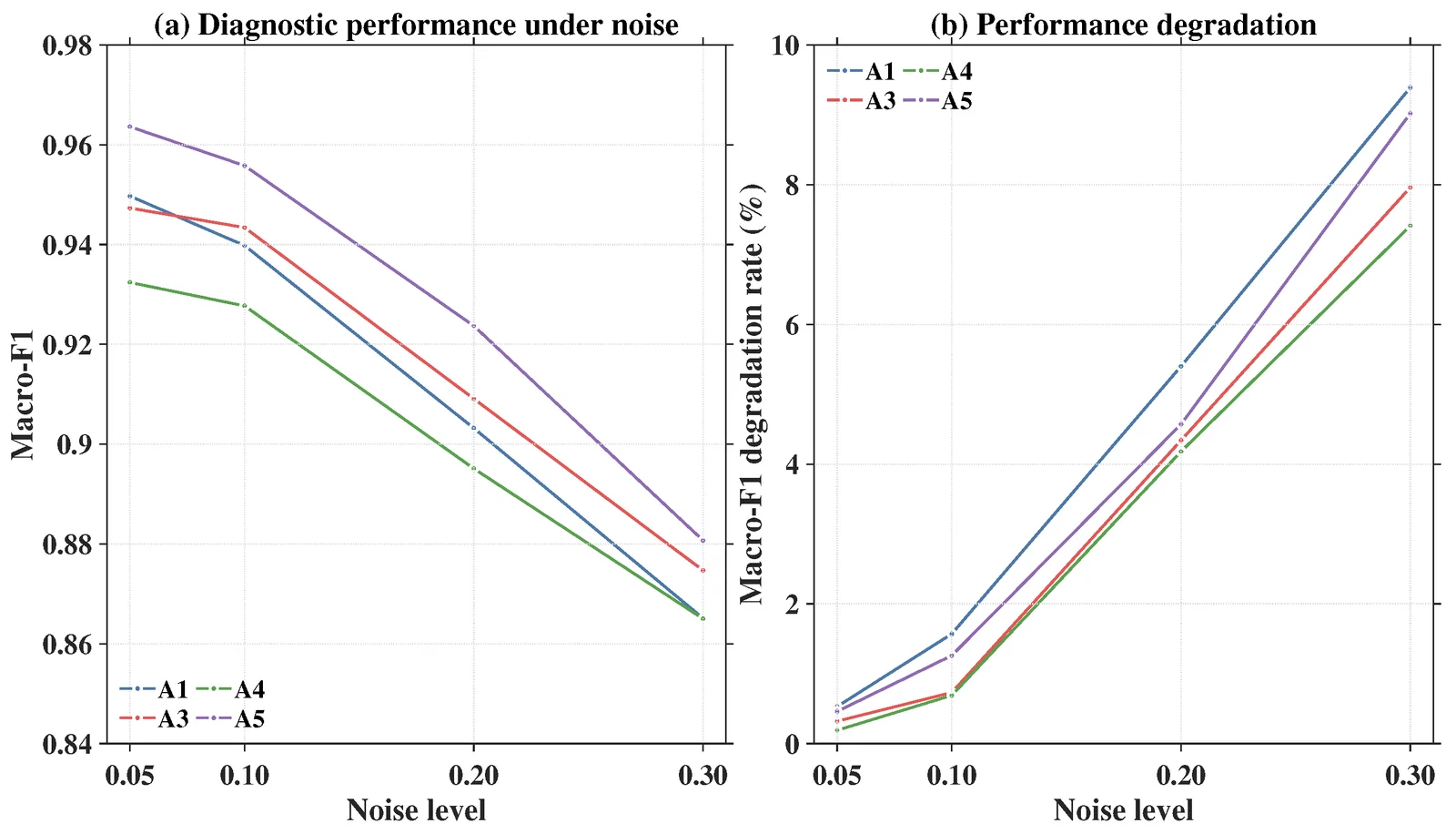
Macro-F1 and relative degradation under feature noise on the SEU gearbox dataset.

Here, Acc(0) and Macro-F1(0) denote the results obtained by the same model under noise-free conditions. A lower relative degradation rate indicates that the model is less sensitive to input noise. Table 21 reports the Accuracy of each configuration at different noise levels. Figure 11 presents the Macro-F1 values and their relative degradation rates, respectively. The noise-free result is used as the baseline for calculating the degradation rate; therefore, the degradation curves start from a noise level of 0.05.

As shown in Table 21, the Accuracy of all four evaluated configurations (A1, A3, A4, and A5) decreases gradually as the noise level increases. A5 achieves the highest absolute Accuracy at all five noise levels, decreasing from 96.81% under noise-free conditions to 88.08% at a noise level of 0.30. This result indicates that the complete HSD-BRB retains a strong fault recognition capability when the gearbox features are perturbed [27,28]. At a noise level of 0.30, A3 reaches an Accuracy of 87.48%, slightly higher than those of A1 and A4, but remains below A5.

Figure 11a shows that A5 retains the highest Macro-F1 across the tested feature-noise levels. In Figure 11b, relative degradation increases with noise for every configuration, but A4 has the smallest decline, reaching 7.41% at a noise level of 0.30. Thus, A5 provides the strongest absolute performance in this experiment, while A4 is less sensitive in relative terms.

### 4.5. Raw-Vibration Noise Robustness

The preceding experiment adds Gaussian noise directly to standardized features. To assess the effect of noise before feature extraction, a separate test added zero-mean Gaussian noise to each held-out 4096-point gearbox vibration segment at signal-to-noise ratios (SNRs) of 30, 20, 10, and 0 dB. Noise power was set independently for each segment. The same six features were then re-extracted and standardized using the unchanged training-set parameters before inference with the archived A1, A3, A4, and A5 models. The original chronological train/test partition, test labels, and BRB reference values were retained; no model was retrained. The reconstructed noise-free features were checked against the stored test features before evaluation. Each SNR was evaluated with three archived model runs and matched noise realizations across configurations.

Performance changes little at 30 dB (Table 22). At 20 dB, A5 has the highest mean Accuracy (83.55%) and Macro-F1 (0.8226) among the four configurations, down from 96.80% and 0.9679 without noise. At 10 and 0 dB, its Accuracy falls to 37.06% and 22.08%, respectively. A4 has the highest mean Accuracy at 10 dB, with a large standard deviation of 14.29 percentage points. The sharp deterioration under severe noise marks a practical limit of the feature extraction and diagnosis pipeline. These descriptive comparisons combine variation from the archived models and noise realizations.

### 4.6. Out-of-Range Feature Stress Test

A synthetic feature-shift stress test was conducted using the archived A5 model from the first gearbox run, without retraining. The Energy, Impact, and Spectrum feature groups were shifted separately or jointly in positive and negative directions by 10%, 20%, and 30% of each feature’s range in the original training partition. The original test labels were retained for evaluation. This procedure measures sensitivity to controlled shifts in extracted features; it does not reproduce vibration signals measured under new operating conditions. The unperturbed Accuracy of this single archived run is 97.14%, rather than the ten-run mean of 96.81% reported in Table 17.

As shown in Table 23, simultaneous shifts of all six features substantially reduce diagnostic performance. At a 10% shift, Accuracy falls to 72.21% in the negative direction and 75.58% in the positive direction; at 30%, the corresponding values are 22.86% and 37.66%. Shifting only one semantic group generally causes less degradation than shifting all groups together. The model continues to produce predictions, but the results do not support robust fault classification under large simultaneous feature shifts.

The BRB matching function clips values outside the attribute reference bounds to the nearest endpoint. For every tested shift, predictions from the original perturbed inputs were identical to those from explicitly endpoint-clipped inputs; additional endpoint probes likewise showed no change in fault beliefs beyond a reference endpoint. This ensures that rule matching remains defined but removes information about the magnitude of further exceedance. The reference bounds are narrower than the full training-data ranges: even without perturbation, 64.16% of test samples exceed at least one reference bound, whereas 1.56% exceed a training-set minimum or maximum. Therefore, reference-bound exceedance should not be equated with an unseen operating condition. Because the shifts were applied to extracted features while retaining the original labels, this test does not establish generalization to actual changes in speed or load.

The degradation in Section 4.5 and Section 4.6 can be interpreted through the feature-to-semantic-to-rule inference pipeline. Noise in the measured vibration signal first perturbs the extracted statistical and spectral features, whereas the feature-shift experiment introduces displacement directly at the feature input. These changes alter the Low, Medium, and High membership degrees and the resulting semantic-state beliefs. Beyond an attribute’s reference bounds, endpoint clipping removes information about the magnitude of further displacement. Changes in the semantic beliefs then propagate to the top-level rule activations and final fault beliefs. The observed degradation is consistent with this propagation mechanism. In practice, signal-quality checks can help identify unreliable measurements before feature extraction, while monitoring reference-bound exceedance and semantic memberships can reveal changes in the input conditions.

The noise experiments show tolerance to modest noise, while severe noise and simultaneous feature shifts cause substantial degradation. Under such conditions, recalibration of feature references or additional training data from the relevant operating conditions should be considered before deployment.

### 4.7. Hyperparameter Sensitivity Analysis

A one-factor-at-a-time sensitivity analysis was conducted on the SEU gearbox dataset using the original training/test partition and the same stratified 10% student-training subset. The classification and semantic teachers were held fixed. The default settings were T=2, η=0.1, $\lambda_{s}=0.2$, $\lambda_{p}=0.1$, and $\lambda_{e}=0.02$. Each setting was evaluated using three matched initialization runs, with all other parameters unchanged. Each fit used the same SQP budget of 150 iterations and 40,000 objective-function evaluations.

Table 24 shows that the model is most sensitive to the temperature *T*. Increasing *T* from 2 to 4 reduces mean Accuracy from 96.6234% to 86.4069% and also increases both State-KL and Path-KL. In contrast, reducing *T* to 1 produces only a moderate decrease in Accuracy to 95.4978% while yielding lower State-KL and Path-KL. This indicates that excessive softening of the teacher distribution can weaken the class-discriminative information transferred to the BRB, whereas a lower temperature places greater emphasis on the dominant teacher predictions.

The uncertainty-mixing coefficient η shows a more stable response. Removing the uniform component (η=0) reduces Accuracy to 93.7662%, whereas η=0.1 and η=0.2 produce very similar classification performance. The increase from η=0.1 to 0.2 slightly improves Accuracy and Macro-F1 and also reduces Path-KL from 1.2425 to 1.1574, suggesting that a moderate amount of uncertainty mixing helps maintain a useful distributional target without substantially weakening class discrimination.

The state-distillation weight $\lambda_{s}$ has a relatively limited effect on final classification performance within the tested range. The highest Accuracy is obtained at $\lambda_{s}=0.1$ (96.9697%), while the default $\lambda_{s}=0.2$ gives 96.6234%. As $\lambda_{s}$ increases from 0.05 to 0.1 and 0.2, State-KL decreases monotonically from 0.2421 to 0.2074 and 0.2004. Stronger state supervision therefore improves state-level agreement over these settings, while Accuracy peaks at the intermediate value. The path-distillation weight $\lambda_{p}$ shows a clearer trade-off: increasing $\lambda_{p}$ to 0.2 lowers Path-KL to 0.7270, the smallest value in this sensitivity study, but reduces Accuracy to 94.8918%. Thus, stronger path-level alignment can improve agreement with the teacher path distribution at the expense of final fault classification.

The expert-deviation weight $\lambda_{e}$ produces comparatively small changes over the tested range. Increasing $\lambda_{e}$ from 0.02 to 0.04 raises Accuracy from 96.6234% to 97.0563% and Macro-F1 from 0.9661 to 0.9705, while the State-KL and Path-KL values remain close to those of the default setting. The results therefore suggest that the tested range of $\lambda_{e}$ provides relatively stable behavior, whereas the temperature and path-distillation weight exert stronger effects on the overall objective.

Overall, the sensitivity analysis identifies *T* and $\lambda_{p}$ as the most influential parameters among the tested settings. The default configuration does not maximize every individual metric, but it remains within the high-performing region of the tested parameter ranges and avoids the pronounced classification degradation observed at T=4 and $\lambda_{p}=0.2$. The results also show that classification performance and reasoning-consistency metrics do not change monotonically together, supporting the use of multiple criteria when selecting the loss weights.

To further examine the sensitivity to the expert-initialized consequent beliefs, we varied the concentration of the initial belief distributions using a power transformation. For the initial consequent belief of the *k*-th rule and the *h*-th state, denoted by $\beta_{k,h}^{0}$, the perturbed belief degree is defined as

(57) $${\tilde{\beta }}_{k,h}\left(p\right)=\frac{{\left(\beta_{k,h}^{0}\right)}^{p}}{\sum_{j=1}^{H}{\left(\beta_{k,j}^{0}\right)}^{p}},\;h=1,\ldots ,H.$$

Here, *p* denotes the belief-concentration factor. The original initialization corresponds to p=1. When p<1, the belief distribution becomes less concentrated, whereas p>1 makes it more concentrated. Because the power transformation is monotonic for nonnegative belief degrees, the ordering of the belief degrees, and hence the highest-belief class of each rule, is preserved. We evaluated p∈{0.8,1.0,1.2}. The original 90 training samples, 385 held-out test samples, teacher targets, and optimization budget were kept fixed. The initial rule weights and attribute weights were also kept unchanged, while the reference beliefs used in the expert-deviation constraint were adjusted consistently with each modified initialization.

As shown in Table 25, Accuracy ranges from 96.3636% to 97.4026%, while Macro-F1 ranges from 0.9636 to 0.9740 across the three belief-concentration settings. Relative to the original initialization (p=1), the Accuracy changes by −0.7792 and +0.2597 percentage points for p=0.8 and p=1.2, respectively, while the corresponding Macro-F1 changes are −0.0078 and +0.0026. These results indicate limited variation in classification performance under the tested changes in initial belief concentration, while they do not imply insensitivity to arbitrary expert-rule initialization.

### 4.8. Computational Cost Analysis

The classification teacher, HSD-BRB, and a fixed-weight flat BRB were benchmarked on the same 385 gearbox test samples under the same MATLAB R2023b and workstation environment. HSD-BRB and the flat BRB were trained using the same stratified 90-sample subset, while the teacher was evaluated using its pretrained model. Table 26 summarizes the model size, training cost, and batch inference time.

HSD-BRB uses 54 rules compared with 729 rules in the flat BRB, reducing the rule count by 92.6%. Its mean batch inference time is 86.5% lower than that of the teacher and 28.2% lower than that of the flat BRB in the tested implementation. This reduction comes with higher training cost because HSD-BRB requires iterative optimization, whereas the flat BRB obtains consequent beliefs directly from activation-weighted class counts. The results therefore show a computational trade-off: the hierarchical structure substantially reduces the rule space and measured inference cost while increasing optimization cost during training.

The smaller reduction in inference time (28.2%) than in rule count (92.6%) shows that runtime also depends on other aspects of the implementation. These timings exclude signal acquisition and feature preprocessing. End-to-end latency additionally depends on the 4096-point sensing window, feature extraction, signal transfer, and hardware throughput. Only the trained HBRB is required during inference.

The HSD-BRB training time of 131.7691 s in Table 26 is a fixed-budget measurement under the original solver settings. To examine the effect of further optimization, three single fits were restarted from the same archived initialization using the same 90-sample subset, teacher targets, and loss function. The original SQP limits of 150 iterations and 40,000 function evaluations were increased by factors of two and four, with all tolerances unchanged. Table 27 reports the results.

The 131.7691 s reported in Table 26 is the original standalone benchmark measurement, whereas the 126.0845 s reported for the 1× budget in Table 27 comes from a separate rerun performed for the solver-budget sensitivity analysis. Both runs use the same nominal solver budget and experimental settings, but they were executed separately; therefore, their wall-clock times are not expected to be identical. Table 27 is intended to characterize the effect of increasing the solver budget rather than to reproduce the original benchmark timing. Increasing the budget lowers the final loss from 0.2416 to 0.1824, while training time rises from 126.0845 s to 479.9950 s. Test Accuracy changes from 97.1429% to 95.5844%, and Macro-F1 from 0.9714 to 0.9555. Further objective minimization does not translate into improved held-out classification in this comparison.

### 4.9. Experimental Summary

In the main bearing ablation, Accuracy increases from 88.05% in A1 to 95.58% in A2 with output and semantic-state distillation and to 96.28% in A3 with path distillation. The complete configuration records 95.76% with the expert constraint. On the gearbox dataset, A5 records 96.81% Accuracy and 0.9680 Macro-F1 using 90 labelled samples and privileged supervision from a teacher trained on the full training partition. However, the ten-run paired tests in Table 18 find no statistically significant Accuracy or Macro-F1 difference between A5 and A1, A3, or A4 after Holm correction. The main-experiment ranking therefore does not establish classification superiority of the complete configuration. The reported consistency measures describe a separate trade-off: A5 has the lowest State-KL in the main gearbox experiment, whereas A4 has lower Path-KL. The diagnostic cases illustrate how semantic beliefs and activated rules contribute to individual predictions.

The additional experiments define the practical trade-offs. On the bearing dataset, HSD-BRB outperforms the fixed-weight Flat-BRB but falls below the SVM, Random Forest, and ANFIS baselines in classification accuracy. The gearbox implementation uses fewer rules and has lower measured batch inference cost than the flat reference, at the expense of iterative training. Performance tolerates modest signal noise but deteriorates under severe noise and large feature shifts.

### 4.10. Practical Limitations

Several limitations should be considered when interpreting these results. The semantic states are defined from feature groups and training-set reference values, so they represent operational targets rather than independently validated physical quantities. In the gearbox small-sample experiment, the teacher uses the complete training partition while the BRB student is optimized with 90 labelled samples, corresponding to a privileged-supervision setting. The robustness experiments cover controlled feature shifts and signal perturbations rather than the full range of changes in speed, load, sensor placement, and long-term degradation. In addition, BRB optimization is computationally nonconvex, and convergence was not confirmed for every fit under the fixed solver budgets used in the supplementary experiments.

The expert initialization also depends on the selected feature grouping, fault prototypes, and qualitative rule tendencies. The present study evaluates the sensitivity of the initial consequent belief concentration within the tested range, but does not systematically evaluate broader alternative expert priors or changes to the underlying feature grouping and qualitative rule structure. The listed initial belief degrees should therefore be interpreted as structured expert-guided priors rather than unique or universally optimal assessments.

The current scalability analysis is limited to six extracted features organized into the expert-defined Energy, Impact, and Spectrum groups. The 54-rule hierarchical structure is substantially smaller than the 729-rule flat reference in the tested configuration, but extending HSD-BRB to raw vibration signals, spectrograms, or high-dimensional learned features would require additional feature organization and semantic-state design. Hierarchical decomposition reduces the rule count in the present configuration but does not remove combinatorial growth as the number of attributes, reference states, or semantic branches increases. The current experiments therefore establish the behavior of HSD-BRB for the tested feature representation rather than for arbitrary high-dimensional inputs.

## 5. Conclusions

HSD-BRB transfers diagnostic outputs, semantic-state distributions, and rule-path targets from neural teachers to a hierarchical belief rule base. The resulting model provides fault predictions together with inspectable intermediate beliefs and rule activations. In the main bearing and gearbox experiments, the complete configurations record mean accuracies of 95.76% and 96.81%, respectively. The supplementary gearbox paired tests do not establish a statistically significant classification advantage of A5 over A1, A3, or A4 under the fixed optimization budget. The complete configuration should therefore be viewed as a combination of classification, teacher-alignment, and expert-preservation objectives, rather than as a uniformly superior classifier. The experiments demonstrate the feasibility of this multilevel transfer mechanism for the tested feature representations, while showing that classification accuracy and reasoning consistency do not necessarily improve together.

The method is intended for diagnosis from monitoring features with available domain knowledge. Its use under variable operating conditions and with larger feature sets requires further validation, as discussed in Section 4.10.

Future work will focus on three directions. The first is adaptive semantic modeling for variable-condition and cross-device applications, with the aim of reducing dependence on manually defined groups and fixed expert priors. The second is the development of more efficient rule-parameter optimization and incremental updating methods for large-scale attributes and online diagnosis. The third is to improve uncertainty quantification, rejection of unreliable samples, and human–machine verification, so that the model can alert engineers when the diagnostic evidence is insufficient and provide greater value in practical maintenance systems.

## Figures and Tables

**Table 1:** Representative related studies and their relationship to the present research questions.

Study/Strand	Established Contribution	Question Addressed by HSD-BRB
Shao et al. [6]; data-driven diagnosis	Deep feature learning and transfer learning for machinery fault recognition.	How can a student expose semantic states and rule activations in addition to a fault label?
Dou et al. [7]; rule-based diagnosis	Signal-feature extraction, decision-table rule induction, and explicit rule matching.	How can teacher supervision be associated with hierarchical belief distributions?
Cao et al. (2021) [10]; approximate BRB	Approximate BRB construction for interpretable complex-system modeling.	How can intermediate student states be aligned with specified teacher targets?
Hu et al. [11]; hierarchical BRB	Hierarchical modeling with feature selection for imbalanced multiclass problems.	How can state supervision and top-level path supervision be combined?
Yang et al. (2023) [12]; tree-structured BRB	A multilayer tree structure for complex-problem modeling.	How can knowledge transfer be evaluated at different reasoning levels?
Ming et al. [13]; BRB-P	Probability-table initialization and interpretability-constrained optimization.	How can expert preservation be assessed alongside teacher–student alignment?
Cao et al. (2024) [8]; interpretable H-BRB	Readability-oriented construction and consistency-preserving optimization.	How can named intermediate states support an inspectable distillation process?
Zeng et al. [17]; distillation-enhanced BRB	Discrepancy identification, weighted prediction alignment, and contribution analysis.	How can output alignment be supplemented by explicit semantic-state and rule-path targets?

Studies are cited in the preceding paragraphs. The final column identifies the focus of this work, not a claim that the earlier methods are unsuitable for their original tasks.

**Table 2:** Main experimental configurations for the SEU bearing and gearbox datasets.

Setting	Bearing	Gearbox
Nominal speed-frequency setting (Hz)	20	30
Load-voltage setting (V)	0	2
Original train/test partition	890/385 samples (70%/30%)	890/385 samples (fixed original partition)
Classification teacher data	Full original training partition	Full original training partition; internal 85%/15% fit/validation split
BRB student training data	Full original training partition (890 samples)	Fixed stratified subset of 90 samples (18 per class)
Evaluation data	Original held-out test partition (385 samples)	Original held-out test partition (385 samples)
Input and semantic groups	6 standardized features; Energy, Impact, Spectrum (2 features each)	6 standardized features; Energy, Impact, Spectrum (2 features each)
BRB structure	Three 9-rule sub-BRBs; one 27-rule top-level BRB	Three 9-rule sub-BRBs; one 27-rule top-level BRB
Attribute reference values	Training-set 10th, 50th, and 90th percentiles	Training-set 10th, 50th, and 90th percentiles
Complete-model loss settings	$\lambda_{y}=1$, $\lambda_{o}=0.5$, $\lambda_{s}=0.2$, $\lambda_{p}=0.1$, $\lambda_{e}=0.02$; T=2, η=0.1	$\lambda_{y}=1$, $\lambda_{o}=0.5$, $\lambda_{s}=0.2$, $\lambda_{p}=0.1$, $\lambda_{e}=0.02$; T=2, η=0.1

**Table 3:** Fault classes and label encoding of the SEU bearing dataset.

Label	Fault State	Description
1	Health	Healthy condition
2	Inner	Inner-race fault
3	Outer	Outer-race fault
4	Ball	Rolling-element fault
5	Compound	Compound inner–outer race fault

**Table 4:** Semantic grouping of input attributes for bearing fault diagnosis.

Sub-BRB	Input Attributes	Output States	Grouping Rationale
Energy-BRB	RMS, Variance	Low, Medium, High energy	Both attributes characterize vibration amplitude and overall energy.
Impact-BRB	Kurtosis, Crest Factor	Weak, Medium, Strong impact	Both attributes reflect transient impacts caused by local damage.
Spectrum-BRB	Spectral Centroid, Spectral Bandwidth	Low, Medium, High composite spectral state	Both attributes describe spectral-center shifts and energy dispersion.

**Table 5:** Diagnostic results on the SEU bearing dataset.

Configuration	Model Components	Accuracy	Macro-F1
Teacher	Six-feature DNN teacher (single training run; reference only)	97.9221%	0.9791
A1	Label supervision only	88.05±1.04%	0.8775±0.0114
A2	Output and semantic-state distillation	95.58±1.19%	0.9558±0.0120
A3	A2 + diagnostic-path distillation	96.28±0.40%	0.9627±0.0041
A4 (HSD-BRB)	A3 + expert-deviation constraint	95.76±0.60%	0.9575±0.0061

**Table 6:** External baseline comparison on the SEU bearing dataset.

Model	Accuracy (%)	Macro-F1
Flat BRB (fixed weights)	92.2080	0.9212
RBF-SVM	99.1950	0.9919
Random forest	98.3900	0.9839
ANFIS (one-versus-rest)	98.9610	0.9896
DNN teacher (reference)	97.9221	0.9791
HSD-BRB (A4, complete)	96.0520	0.9604

**Table 7:** Paired significance tests for HSD-BRB against external baselines on the SEU bearing dataset.

Comparator	Metric	Mean Difference	95% CI	Paired *t*-Test *p*	Wilcoxon *p*
Flat BRB	Accuracy	+3.8442	[3.5843,4.1040]	<0.001	0.0156
Flat BRB	Macro-F1	+0.0392	[0.0366,0.0419]	<0.001	0.0156
RBF-SVM	Accuracy	−3.1429	[−3.3975,−2.8882]	<0.001	0.0156
RBF-SVM	Macro-F1	−0.0315	[−0.0341,−0.0289]	<0.001	0.0156
Random forest	Accuracy	−2.3377	[−2.6146,−2.0607]	<0.001	0.0156
Random forest	Macro-F1	−0.0235	[−0.0263,−0.0207]	<0.001	0.0156
ANFIS-OVR	Accuracy	−2.9091	[−3.1689,−2.6493]	<0.001	0.0156
ANFIS-OVR	Macro-F1	−0.0292	[−0.0318,−0.0265]	<0.001	0.0156

Mean differences are computed as HSD-BRB minus the comparator. Accuracy differences are in percentage points. The reported *p*-values are Holm-adjusted values; ten matched runs were used for each comparison.

**Table 8:** Single-run comparison of hierarchical and fully optimized flat BRBs under a common loss on the SEU bearing dataset.

Model	Rules	Variables	Accuracy (%)	Macro-F1	Training Time (s)
Hierarchical BRB (common loss)	54	279	88.5714	0.8849	938.43
Fully optimized flat BRB	729	4380	87.5325	0.8732	1778.21

Variables denote packed optimization entries, not independent degrees of freedom. The hierarchical configuration uses the common loss and is distinct from the complete A4 configuration in the main experiment.

**Table 9:** State and path consistency on the SEU bearing dataset.

Configuration	State-KL	Path-KL
A1	1.7762	5.5579
A2	0.2139	1.5974
A3	0.1715	0.7528
A4	0.1697	0.7481

**Table 10:** Case-level diagnostic traces.

Item	Case 1	Case 2
Test sample	No. 78	No. 1
True class	Inner	Health
Predicted class	Inner	Health
Energy-state belief {L,M,H}	{0.0016,0.8289,0.1694}	{0.0004,0.1149,0.8847}
Impact-state belief {L,M,H}	{0.1000,0.8809,0.0190}	{0.9995,0.0005,0.0000}
Spectrum-state belief {L,M,H}	{0.0783,0.9212,0.0004}	{0.0821,0.1021,0.8159}
Dominant-state combination	(M,M,M)	(H,L,H)
Dominant rule	$R_{14}$	$R_{21}$
Rule activation	0.8711	0.9542
Final fault belief	{0.0041,0.9876,0.0005,0.0055,0.0022}	{0.9945,0.0030,0.0003,0.0006,0.0015}

Note: The final fault-belief vector is ordered as {Health, Inner, Outer, Ball, Compound}. The labels L, M, and H denote group-specific operational states defined using training-set reference values, not fault-severity levels. The final fault belief is obtained by rule aggregation and is not the consequent belief of the dominant rule alone.

**Table 11:** Mean absolute Shapley attributions over the 25 selected bearing test samples.

Feature	Teacher	HSD-BRB (A4)
RMS	0.0062	0.1787
Variance	0.0002	0.1888
Kurtosis	0.1450	0.0832
Crest factor	0.0311	0.0109
Spectral centroid	0.5363	0.1800
Spectral bandwidth	0.1165	0.1144

**Table 12:** Local surrogate fit and coefficient variability for the bearing models.

Metric	Teacher	HSD-BRB (A4)
Weighted $R^{2}$	0.3399±0.2320	0.3474±0.1669
Weighted MAE	0.2268±0.1096	0.2739±0.0645
Coefficient variability	0.0162±0.0065	0.0182±0.0040

**Table 13:** Fault classes and label encoding of the SEU gearbox dataset.

Label	Fault State	Description
1	Health	Healthy gearbox
2	Chipped	Gear-tooth chipping
3	Miss	Missing tooth
4	Root	Gear-root fault
5	Surface	Gear-surface fault

**Table 14:** Teacher and student performance with different teacher training data sizes on the SEU gearbox dataset.

Model or Student Configuration	Teacher Samples	Accuracy (%)	Macro-F1
Classification teacher	90	98.7013±0.9365	0.9870±0.0094
Classification teacher	890	99.0476±0.2999	0.9905±0.0030
Label supervision + expert constraint	–	96.9697±0.3968	0.9696±0.0040
Output distillation	90	97.2294±0.2999	0.9722±0.0030
Output distillation	890	96.8831±0.5195	0.9688±0.0052
Complete distillation	90	96.9697±0.1500	0.9697±0.0015
Complete distillation	890	96.7965±0.3968	0.9679±0.0040

Results are mean ± standard deviation over three repetitions. All students use 90 labelled samples. Teacher sample counts include the internal fit and validation partitions. Every student configuration includes the same expert constraint ($\lambda_{e}=0.02$). Complete distillation adds output, state, and path losses.

**Table 15:** Comparison of semantic supervision sources on the SEU gearbox dataset using the complete training partition (single run).

Semantic Teacher	Teacher State Accuracy (%)	Student Accuracy (%)	Student Macro-F1	State-KL	Path-KL
Full-input MLP	97.4890	97.6620	0.9766	0.2186	0.6761
Quantile mapping	100.0000	97.6620	0.9766	0.2656	0.8857
Subgroup-only MLP	98.5280	96.8830	0.9688	0.2544	0.8231

**Table 16:** Effect of state distillation under different semantic teachers on the SEU gearbox dataset.

Semantic Teacher	State-KL		Accuracy (%)		Macro-F1	
	Off	On	Off	On	Off	On
Full-input MLP	0.3255	0.2013	96.5370	96.1900	0.9651	0.9618
Quantile mapping	0.4375	0.2554	96.5370	96.1040	0.9651	0.9609
Subgroup-only MLP	0.3309	0.2073	96.5370	96.7100	0.9651	0.9670

Values are means over three paired runs. Off and On denote state distillation disabled and enabled, respectively.

**Table 17:** Diagnostic performance and consistency results on the SEU gearbox dataset.

Configuration	Model Components	Accuracy (%)	Macro-F1	State-KL	Path-KL
Teacher	Six-feature DNN (full training partition; reference)	99.48	0.9948	–	–
A0	No distillation	92.18 ± 5.73	0.9185 ± 0.0573	5.4148±1.9381	17.2056±4.3770
A1	Label supervision	95.53 ± 2.40	0.9547 ± 0.0240	0.5839±0.1467	2.5629±0.6182
A2	Output distillation	95.30 ± 1.71	0.9528 ± 0.0171	0.4378±0.2276	2.4279±0.7020
A3	Output + state distillation	95.06 ± 3.05	0.9504 ± 0.0305	0.2656±0.2276	2.1024±0.7020
A4	A3 + path distillation	93.45 ± 4.68	0.9342 ± 0.0468	0.2116±0.0227	1.1093±0.2309
A5	Complete HSD-BRB	96.81 ± 0.37	0.9680 ± 0.0037	0.2091±0.0279	1.2486±0.3068

**Table 18:** Paired significance tests comparing A5 with the gearbox ablation configurations.

Comparison	Metric	Mean Difference	95% CI	$p_{t}$	$p_{w}$	Holm-Adjusted *p*
A5–A1	Accuracy	−0.8831	[−4.0705,2.3043]	0.5464	0.5703	1.0000
A5–A1	Macro-F1	−0.0087	[−0.0415,0.0242]	0.5652	0.4922	1.0000
A5–A3	Accuracy	2.0260	[−1.8118,5.8638]	0.2629	0.3887	1.0000
A5–A3	Macro-F1	0.0207	[−0.0182,0.0596]	0.2598	0.3750	1.0000
A5–A4	Accuracy	0.2338	[−0.2334,0.7010]	0.2869	0.4844	1.0000
A5–A4	Macro-F1	0.0023	[−0.0024,0.0071]	0.2969	0.4609	1.0000

$p_{t}$ and $p_{w}$ denote the two-sided paired *t*-test and Wilcoxon signed-rank test *p*-values, respectively. Mean differences are computed as A5 minus the comparator; Accuracy differences are in percentage points.

**Table 19:** Spearman correlations among semantic-state scores on the held-out test sets.

Dataset	Energy–Impact	Energy–Spectrum	Impact–Spectrum
Bearing	−0.3531	0.1420	0.2964
Gearbox	0.0002	−0.8553	0.2077

**Table 20:** Factorized and joint path-target constructions on the SEU gearbox dataset.

Path-Target Construction	Accuracy (%)	Macro-F1	State-KL
Original main experiment (ten-run mean; reference only)			
Complete HSD-BRB with factorized product	96.8100	0.9680	0.2091
Additional single matched comparison			
Factorized product	87.7922	0.8769	0.2254
Joint auxiliary network	89.3506	0.8924	0.2050

The ten-run main-experiment row is provided for context and is not paired with either row in the additional comparison. The two rows under the additional comparison were initialized identically and use a common limit of 200 iterations and 50,000 objective-function evaluations.

**Table 21:** Accuracy under different noise levels on the SEU gearbox dataset.

Noise Level	A1	A3	A4	A5
0.00	95.53%	95.06%	93.45%	96.81%
0.05	95.04%	94.75%	93.27%	96.36%
0.10	94.05%	94.36%	92.83%	95.58%
0.20	90.49%	90.96%	89.53%	92.39%
0.30	86.68%	87.48%	86.52%	88.08%

**Table 22:** Accuracy under additive noise applied to the raw gearbox vibration signals.

SNR (dB)	A1 (%)	A3 (%)	A4 (%)	A5 (%)
No noise	95.41±1.33	96.36±0.26	93.68±5.78	96.80±0.60
30	95.24±1.31	96.28±0.54	93.68±5.33	96.80±0.15
20	81.30±0.45	82.60±0.52	79.83±3.21	83.55±0.54
10	39.31±4.68	41.73±10.89	46.06±14.29	37.06±3.49
0	30.74±0.83	21.04±3.16	21.82±2.50	22.08±1.19

Values are mean ± sample standard deviation over three archived model runs, each paired with one noise realization per SNR. SNR is defined at the raw-signal level and is not numerically interchangeable with the feature-noise levels in Table 21.

**Table 23:** Gearbox A5 performance under simultaneous shifts of all six input features.

Relative Shift	Accuracy (%)	Macro-F1	Prediction Agreement	Outside Training Range (%)
None	97.14	0.9714	1.0000	1.56
−10%	72.21	0.7193	0.7247	20.00
+10%	75.58	0.7441	0.7610	11.95
−20%	57.92	0.5845	0.5766	54.81
+20%	49.87	0.4290	0.5039	40.00
−30%	22.86	0.2593	0.2286	95.32
+30%	37.66	0.2598	0.3896	66.49

Prediction agreement is the fraction of labels unchanged from the unperturbed A5 run. Outside training range is the percentage of test samples with at least one feature beyond the original training minimum or maximum.

**Table 24:** Hyperparameter sensitivity on the SEU gearbox dataset.

Parameter	Value	Accuracy (%)	Macro-F1	State-KL	Path-KL
*T*	1	95.4978±0.3968	0.9548±0.0040	0.1704	0.8681
	2 *	96.6234±0.6872	0.9661±0.0069	0.2004	1.2425
	4	86.4069±4.5757	0.8635±0.0460	0.2724	1.3736
η	0	93.7662±3.6364	0.9375±0.0364	0.2219	1.2665
	0.1 *	96.6234±0.6872	0.9661±0.0069	0.2004	1.2425
	0.2	96.7100±0.3968	0.9670±0.0040	0.2009	1.1574
$\lambda_{s}$	0.05	96.1905±1.0497	0.9617±0.0107	0.2421	1.1041
	0.1	96.9697±0.2999	0.9696±0.0030	0.2074	1.2384
	0.2 *	96.6234±0.6872	0.9661±0.0069	0.2004	1.2425
$\lambda_{p}$	0.05	96.3636±0.2597	0.9635±0.0026	0.1994	1.3772
	0.1 *	96.6234±0.6872	0.9661±0.0069	0.2004	1.2425
	0.2	94.8918±1.5219	0.9489±0.0151	0.2122	0.7270
$\lambda_{e}$	0.01	96.9697±0.1500	0.9696±0.0015	0.2069	1.2497
	0.02 *	96.6234±0.6872	0.9661±0.0069	0.2004	1.2425
	0.04	97.0563±0.1500	0.9705±0.0015	0.2024	1.2292

Accuracy and Macro-F1 are reported as mean ± sample standard deviation over three runs; State-KL and Path-KL are means. An asterisk marks the default value. The five default rows repeat the same three baseline runs and do not represent additional independent observations.

**Table 25:** Sensitivity to the initial consequent belief concentration factor *p* on the SEU gearbox dataset.

Belief-Concentration Factor *p*	Accuracy (%)	Macro-F1
0.8	96.3636	0.9636
1.0	97.1429	0.9714
1.2	97.4026	0.9740

**Table 26:** Computational cost on the gearbox dataset using a batch of 385 test samples.

Model	Rules	Parameter Entries	Training Time (s)	Batch Inference (ms)	Batch Time/Sample (μs)
Teacher	–	2693	–	85.3625±66.2513	221.7208±172.0814
HSD-BRB	54	279	131.7691	11.5170±2.3407	29.9142±6.0797
Flat BRB (fixed weights)	729	3645	0.0868	16.0443±3.8258	41.6735±9.9371

Parameter entries count 2693 trainable network coefficients for the teacher, 279 packed optimization variables for HSD-BRB, and 729×5=3645 consequent belief entries for the flat BRB. The flat BRB has fixed rule and attribute weights, and each consequent vector sums to one. These counts describe stored or optimized entries, not a common measure of independent degrees of freedom. Inference times are mean ± standard deviation over five repetitions after warm-up. Time per sample is batch time divided by 385, not independently measured single-sample latency. Training times are single benchmark measurements; teacher training and target preparation are excluded from the HSD-BRB training time.

**Table 27:** Effect of solver budget on HSD-BRB training and test performance on the SEU gearbox dataset.

Budget	Final Loss	Accuracy (%)	Macro-F1	Training Time (s)
1×	0.2416	97.1429	0.9714	126.0845
2×	0.1935	95.8442	0.9582	253.1413
4×	0.1824	95.5844	0.9555	479.9950

Each row is one fit from the same starting point. Times are newly measured wall-clock durations and are separate from the original benchmark timing.

## Data Availability

The SEU rotating machinery datasets used in this study are publicly available at https://github.com/Yxz3930/SEU-datasets (accessed on 27 August 2026).

## Nomenclature

Acronym	Full name
BRB	Belief Rule Base
HBRB	Hierarchical Belief Rule Base
HSD-BRB	Hierarchical Semantic Knowledge-Distilled Belief Rule Base
KL	Kullback–Leibler
DNN	Deep Neural Network
RMS	Root Mean Square
SEU	Southeast University
RIMER	Rule-base Inference Methodology using Evidential Reasoning
MLP	Multilayer Perceptron
SNR	Signal-to-Noise Ratio
KD	Knowledge Distillation
ER	Evidential Reasoning
SVM	Support Vector Machine
RBF	Radial Basis Function
ANFIS	Adaptive Neuro-Fuzzy Inference System
OVR	One-versus-Rest
SHAP	SHapley Additive exPlanations
LIME	Local Interpretable Model-Agnostic Explanations
SQP	Sequential Quadratic Programming
MAE	Mean Absolute Error
Main Symbols	
Symbol	Description
*N*	Number of samples
$x_{i},y_{i},{\hat{y}}_{i}$	Input vector, true class, and predicted class of sample *i*
*M*	Number of input attributes
*C*	Number of fault classes
*Q*	Number of semantic subspaces (sub-BRBs)
$L_{q}$	Number of rules in the *q*-th sub-BRB
$L_{T}$	Number of rules in the top-level BRB
$H_{q}$	Number of intermediate states in the *q*-th semantic subspace
$\beta_{n,k}$	Belief degree assigned to state *n* by rule *k*
$\theta_{k}$	Weight of rule *k*
$\delta_{m}$	Weight of attribute *m*
$p_{i}^{T}$	Teacher fault probability distribution for sample *i*
$\beta_{i}^{S}$	Student fault belief distribution for sample *i*
$s_{i,q}^{T}$	Teacher semantic-state distribution for subspace *q*, sample *i*
$s_{i,q}^{S}$	Student semantic-state belief distribution for subspace *q*, sample *i*
$\pi_{i}^{T}$	Teacher path-support distribution for sample *i*
$\omega_{i}^{S}$	Student rule-activation distribution for sample *i*
*T*	Temperature parameter for knowledge distillation
η	Uniform uncertainty-mixing coefficient
ε	Small positive numerical constant
$\Theta ,\Theta^{0}$	Current and initial BRB parameters
$A_{q,m,j}$	Reference value for attribute *m* and state *j* in subspace *q*
$M_{q},J_{q,m}$	Numbers of attributes and attribute reference states
$G,\Psi_{q},\Psi_{T}$	Attribute grouping and sub-BRB/top-level initialization mappings
α	Noise or perturbation magnitude, as defined in each experiment
$\sigma_{m}^{\mathrm{tr}}$	Training standard deviation of feature *m*
$D_{\mathrm{KL}}$	Kullback–Leibler divergence
L	Overall training objective
$\lambda_{y},\lambda_{o},\lambda_{s},\lambda_{p},\lambda_{e}$	Loss weights for label, output, state, path, and expert-deviation terms

## Appendix A

This appendix lists the expert-initialized bearing rules constructed using the procedure in Section 3.2. The rule structure and qualitative fault tendencies were formulated by the authors from the physical interpretation of the extracted features and the documented bearing fault characteristics. Table A1 gives the top-level rules, whose belief vectors follow the order Health, Inner, Outer, Ball, and Compound. Table A2, Table A3 and Table A4 give the Energy, Impact, and Spectrum sub-BRB rules, respectively; their belief vectors follow the order Low, Medium, and High. Reference values are calibrated from the training data, and the listed rule weights and consequent beliefs are generated by the reproducible initialization equations provided in Appendix B. They serve as structured prior knowledge and are subsequently adjusted during optimization.

**Table A1:** Initial belief rules of the top-level BRB.

No.	Antecedent	Rule Weight	Belief Degree
1	(L,L,L)	0.9810	{0.958,0.011,0.011,0.011,0.010}
2	(L,L,M)	0.9022	{0.783,0.055,0.067,0.055,0.041}
3	(L,L,H)	0.6597	{0.185,0.201,0.244,0.206,0.164}
4	(L,M,L)	0.7203	{0.378,0.249,0.171,0.161,0.042}
5	(L,M,M)	0.7013	{0.044,0.316,0.336,0.285,0.018}
6	(L,M,H)	0.7088	{0.024,0.272,0.353,0.324,0.028}
7	(L,H,L)	0.7468	{0.032,0.437,0.238,0.261,0.032}
8	(L,H,M)	0.7067	{0.008,0.348,0.289,0.346,0.009}
9	(L,H,H)	0.7296	{0.009,0.284,0.291,0.399,0.017}
10	(M,L,L)	0.8457	{0.657,0.091,0.094,0.086,0.073}
11	(M,L,M)	0.6792	{0.234,0.193,0.287,0.193,0.093}
12	(M,L,H)	0.6941	{0.123,0.210,0.320,0.222,0.125}
13	(M,M,L)	0.7311	{0.045,0.403,0.279,0.245,0.027}
14	(M,M,M)	0.7180	{0.007,0.320,0.373,0.280,0.019}
15	(M,M,H)	0.7176	{0.008,0.258,0.372,0.292,0.069}
16	(M,H,L)	0.7574	{0.013,0.461,0.250,0.262,0.014}
17	(M,H,M)	0.7058	{0.003,0.346,0.303,0.318,0.030}
18	(M,H,H)	0.7009	{0.003,0.259,0.282,0.335,0.121}
19	(H,L,L)	0.6455	{0.190,0.207,0.212,0.203,0.189}
20	(H,L,M)	0.6762	{0.146,0.212,0.280,0.214,0.148}
21	(H,L,H)	0.6699	{0.155,0.203,0.266,0.210,0.165}
22	(H,M,L)	0.7124	{0.059,0.361,0.280,0.240,0.061}
23	(H,M,M)	0.7130	{0.015,0.287,0.362,0.257,0.079}
24	(H,M,H)	0.6839	{0.016,0.194,0.298,0.220,0.273}
25	(H,H,L)	0.7416	{0.030,0.426,0.253,0.254,0.037}
26	(H,H,M)	0.6828	{0.006,0.295,0.278,0.272,0.148}
27	(H,H,H)	0.7506	{0.005,0.160,0.186,0.203,0.446}

The belief-degree vector is ordered as {Health, Inner, Outer, Ball, Compound}.

**Table A2:** Initial belief rules of the Energy-BRB.

No.	Antecedent	Rule Weight	Belief Degree
1	(L,L)	0.9661	{0.915,0.085,0.000}
2	(L,M)	0.8045	{0.511,0.485,0.004}
3	(L,H)	0.9373	{0.087,0.843,0.070}
4	(M,L)	0.8043	{0.485,0.511,0.005}
5	(M,M)	0.9376	{0.078,0.844,0.078}
6	(M,H)	0.8043	{0.005,0.511,0.485}
7	(H,L)	0.9373	{0.070,0.843,0.087}
8	(H,M)	0.8045	{0.004,0.485,0.511}
9	(H,H)	0.9661	{0.000,0.085,0.915}

**Table A3:** Initial belief rules of the Impact-BRB.

No.	Antecedent	Rule Weight	Belief Degree
1	(L,L)	0.9661	{0.915,0.085,0.000}
2	(L,M)	0.8056	{0.514,0.482,0.004}
3	(L,H)	0.9371	{0.089,0.843,0.069}
4	(M,L)	0.8055	{0.482,0.514,0.005}
5	(M,M)	0.9376	{0.078,0.844,0.078}
6	(M,H)	0.8055	{0.005,0.514,0.482}
7	(H,L)	0.9371	{0.069,0.843,0.089}
8	(H,M)	0.8056	{0.004,0.482,0.514}
9	(H,H)	0.9661	{0.000,0.085,0.915}

**Table A4:** Initial belief rules of the Spectrum-BRB.

No.	Antecedent	Rule Weight	Belief Degree
1	(L,L)	0.9661	{0.915,0.085,0.000}
2	(L,M)	0.8050	{0.483,0.512,0.005}
3	(L,H)	0.9372	{0.069,0.843,0.088}
4	(M,L)	0.8051	{0.513,0.483,0.004}
5	(M,M)	0.9376	{0.078,0.844,0.078}
6	(M,H)	0.8051	{0.004,0.483,0.513}
7	(H,L)	0.9372	{0.088,0.843,0.069}
8	(H,M)	0.8050	{0.005,0.512,0.483}
9	(H,H)	0.9661	{0.000,0.085,0.915}

## Appendix B

This appendix provides the detailed parameter initialization procedures that underlie the expert-guided hierarchical semantic modeling described in Section 3.2. The formulas and procedures given here complement the main-text exposition by presenting the complete mathematical derivations for reference-value calibration, belief-degree normalization, rule-weight initialization, and attribute-weight initialization across both sub-BRBs and the top-level BRB.

### Appendix B.1. Reference Value Calibration and Sub-BRB Initialization

For the *m*-th attribute in the *q*-th semantic subspace, define the reference-value set as

(A1) $$\begin{array}{c} A_{q,m}=\left\{A_{q,m,1},A_{q,m,2},\ldots ,A_{q,m,J_{q,m}}\right\}, \end{array}$$

where $J_{\left\{q,m\right\}}$ denotes the number of reference values for the attribute. Different attributes may use different numbers of reference values. For an ordered continuous attribute,

(A2) $$\begin{array}{c} A_{q,m,1}<A_{q,m,2}<\ldots <A_{q,m,J_{q,m}}.\; \end{array}$$

The semantic meanings of the reference values are specified by experts, whereas their numerical locations are calibrated using the training data. Given predefined quantile positions $0<\tau_{q,m,1}<\ldots <\tau_{q,m,J_{q,m}}<1,$ the j-th reference value is obtained as

(A3) $$\begin{array}{c} A_{q,m,j}=Q_{\tau_{q,m,j}}\left({\left\{x_{i,q,m}\right\}}_{i\in D_{\mathrm{tr}}}\right),\; \end{array}$$

where $Q_{\tau }\left(\cdot \right)$ denotes the τ-quantile and $D_{\mathrm{tr}}$ represents the training set. If a physical threshold is known from the domain, it can be directly used as a reference value. The test data are not used to determine the reference values, normalization parameters, or initial rule distributions.

If the *q*-th semantic subspace contains $M_{q}$ input attributes, the number of rules in the corresponding sub-BRB is

(A4) $$\begin{array}{c} L_{q}=\prod_{m=1}^{M_{q}}J_{q,m}.\; \end{array}$$

Each sub-BRB rule corresponds to one combination of attribute reference states. Let $S_{\left\{q,h\right\}}$ denote the *h*-th semantic state of the *q*-th sub-BRB. The initial expert score assigned to state $S_{\left\{q,h\right\}}$ by rule *k* is defined as

(A5) $$\begin{array}{c} u_{q,h,k}^{0}=\Psi_{q}\left(a_{q,k},S_{q,h}\right),\; \end{array}$$

where $a_{q,k}$ denotes the antecedent reference-state combination of rule *k*, and $\Psi_{q}\left(\cdot \right)$ denotes a predefined expert-knowledge mapping. This mapping may be implemented using an expert knowledge table or a parameterized scoring function. Its form and parameters are fixed and recorded during model construction. The expert scoring functions are constrained to produce nonnegative scores, i.e., $\Psi_{q}\left(\cdot \right)\geq 0$ and $\Psi_{T}\left(\cdot \right)\geq 0$.

To avoid zero scores resulting in an invalid normalization, a small positive value ε>0 is introduced. The initial belief degree of rule *k* for state $S_{\left\{q,h\right\}}$ is then given by

(A6) $$\begin{array}{c} \beta_{q,h,k}^{0}=\frac{u_{q,h,k}^{0}+\varepsilon }{\sum_{r=1}^{H_{q}}\left(u_{q,r,k}^{0}+\varepsilon \right)}.\; \end{array}$$

Accordingly,

(A7) $$\begin{array}{c} \sum_{h=1}^{H_{q}}\beta_{q,h,k}^{0}=1.\; \end{array}$$

The consequent belief distributions are initialized using complete belief distributions. Expert uncertainty is represented through the allocation of belief among multiple states rather than by assigning each rule to a single state.

The rule weight is initialized according to the concentration of the consequent belief distribution:

(A8) $$\begin{array}{c} \theta_{q,k}^{0}=\theta_{q,\min}+\left(1-\theta_{q,\min}\right)\max_{h}\beta_{q,h,k}^{0}, \end{array}$$

where $\theta_{q,\min}\in \left[0,1\right]$ is the lower bound of rule reliability for the *q*-th sub-BRB. A more concentrated consequent belief distribution indicates greater confidence in the corresponding expert judgment and therefore results in a higher initial rule weight.

When no sufficient evidence is available to distinguish the relative importance of attributes within a sub-BRB, the attribute weights are initialized uniformly:

(A9) $$\begin{array}{c} \delta_{q,m}^{0}=1,\;m=1,\ldots ,M_{q}.\; \end{array}$$

If domain knowledge provides a reliable ranking of attribute importance, different initial attribute weights may be assigned, provided that their values remain within [0,1].

### Appendix B.2. Top-Level BRB Initialization

After initializing the sub-BRBs, an initial mapping between the intermediate semantic states and the final fault conclusions must be established. Let the semantic-state combination associated with the *l*-th top-level rule be

(A10) $$\begin{array}{c} r_{l}=\left(S_{1,h_{1}\left(l\right)},S_{2,h_{2}\left(l\right)},\ldots ,S_{Q,h_{Q}\left(l\right)}\right).\; \end{array}$$

The initial expert score assigned to fault conclusion $F_{c}$ for this semantic-state combination is defined as

(A11) $$\begin{array}{c} u_{c,l}^{T,0}=\Psi_{T}\left(r_{l},F_{c}\right),\; \end{array}$$

where $\Psi_{T}\left(\cdot \right)$ denotes the top-level expert-knowledge mapping. It describes the initial relationship between different semantic-state combinations and the final fault conclusions. The corresponding consequent belief degree of the top-level rule is

(A12) $$\begin{array}{c} \beta_{c,l}^{T,0}=\frac{u_{c,l}^{T,0}+\varepsilon }{\sum_{r=1}^{C}\left(u_{r,l}^{T,0}+\varepsilon \right)}.\; \end{array}$$

The top-level rule weight is initialized using the same principle as that of the sub-BRBs:

(A13) $$\begin{array}{c} \theta_{l}^{T,0}=\theta_{T,\min}+\left(1-\theta_{T,\min}\right)\max_{c}\beta_{c,l}^{T,0},\; \end{array}$$

where $\theta_{T,\min}$ is the lower bound of the top-level rule reliability. When no clear difference exists among the semantic-state variables, the top-level attribute weights are initialized as

(A14) $$\begin{array}{c} \delta_{T,q}^{0}=1,\;q=1,\ldots ,Q.\; \end{array}$$

After the above initialization, the complete parameter set of the hierarchical BRB can be written as

(A15) $$\begin{array}{c} \Theta^{0}={\left\{\beta_{q}^{0},\theta_{q}^{0},\delta_{q}^{0}\right\}}_{q=1}^{Q}\cup \left\{\beta_{T}^{0},\theta_{T}^{0},\delta_{T}^{0}\right\}.\; \end{array}$$

The corresponding total number of rules is

(A16) $$\begin{array}{c} L_{\mathrm{HBRB}}=\sum_{q=1}^{Q}\left(\prod_{m=1}^{M_{q}}J_{q,m}\right)+\prod_{q=1}^{Q}H_{q}.\; \end{array}$$

The rule-count equation allows different numbers of semantic branches, input attributes, reference values, and intermediate states. It describes the two-level architecture used here; architectures with additional aggregation levels would require a corresponding extension of the construction and rule-count formula.
